# A Scoping Review of Antimicrobial Usage and Antimicrobial Resistance in Beef Cow–Calf Herds in the United States and Canada

**DOI:** 10.3390/antibiotics12071177

**Published:** 2023-07-12

**Authors:** Barbara Wilhelm, Jayce Fossen, Sheryl Gow, Cheryl Waldner

**Affiliations:** 1 Big Sky Health Analytics, Vermilion, AB T9X 2B3, Canada; barbwilhelm16@gmail.com; 2 Department of Large Animal Clinical Sciences, Western College of Veterinary Medicine, University of Saskatchewan, Saskatoon, SK S7N 5B4, Canada; jayce.fossen@usask.ca; 3 Public Health Agency of Canada, Saskatoon, SK S7N 5B4, Canada; sheryl.gow@phac-aspc.gc.ca

**Keywords:** antibiotic use, antibiotic resistance, beef cow, beef calf, antimicrobial susceptibility

## Abstract

Background: The magnitude and knowledge gaps regarding antimicrobial use (AMU) and antimicrobial resistance (AMR) have not been summarized for the North American cow–calf production sector, although estimates of AMU and AMR are essential to AMR risk analysis. The objectives of this scoping review were to map AMU and AMR in the beef cow–calf sector in Canada and the United States, summarize published AMU/AMR predictors, and identify research gaps. Methods: An electronic search was conducted of four bibliographic databases and Google Scholar, augmented by a hand-search of captured studies. Results: Twenty-three of three-hundred and forty-three publications screened advanced to data extraction. Of these, 10 were conducted in the USA and 13 in Canada. Thirteen studied AMR and twelve studied AMU, with two reporting both. Of twelve captured AMU studies, nine presented counts of herd AMU by antimicrobial class or specific antimicrobial. Antimicrobial resistance in *Escherichia coli* (*E. coli*) was reported in nine studies. Risk factors for AMU include herd size, vaccine use, and start date of calving season. Conclusions: Overall, a small number of AMR studies were available for synthesis in primarily one population (cows) reporting *E. coli* AMR. Additional studies targeting reasons for AMU in calves, the impact of management procedures on AMU, potential environmental AMR sources, and AMR in respiratory pathogens and enteric organisms other than *E. coli* for pre-weaning calves are required to inform AMR risk mitigation strategies.

## 1. Introduction

Limiting the emergence and spread of antimicrobial resistance (AMR) is critical to preserving the world’s ability to treat diseases in humans, animals, and plants, reduce food safety and security risks, protect the environment, and maintain progress toward the Sustainable Development Goals [1]. The AMR strategic framework proposed by the World Health Organization (WHO) describes the need for evidence on AMR and antimicrobial use (AMU) which is understandable and available for effective prioritization of AMR by policy-makers [1]. One of the major priorities of this framework is to monitor the AMR burden and AMR response [1].

The use of antimicrobials in livestock production has been identified as one driver of AMR occurrence in both human and animal populations [1]. Antimicrobial resistance in livestock represents both a potential food safety issue and public health concern. Documenting antimicrobial use and resistance is also important in maintaining the social license to continue to produce beef: a recent survey reported that 50% of respondents considered meat labeled “antibiotic-free” to be healthier [2].

The international bodies governing food safety, at both pre-harvest (World Organization of Animal Health (WOAH)) and harvest level (Codex Alimentarius) of the farm-to-fork continuum, recommend risk analysis as the framework for the investigation of animal health and food safety problems to inform policy of member states [3,4]. Estimates of antimicrobial use (AMU) and AMR, and risk factors documented to influence these, are integral to AMR risk analysis [4,5].

While antimicrobials are important to animal health and welfare in all livestock commodities including the beef industry, information is limited on AMU in the North American cow–calf industry. Antimicrobial use has been reported in pre-weaning calves for treatment of diseases such as calf diarrhea and respiratory disease, and in cows for diseases such as lameness associated with infection, although the proportion of individuals treated within a herd tends to be relatively low, in both the United States of America (USA) and Canada [6,7]. These antimicrobials are typically administered by the producer/owner, following instruction from the herd veterinarian, in compliance with the guidelines for the prudent use of antimicrobials drafted by their respective national veterinary associations [8,9]. In Canada, as of December 2018, Health Canada regulatory changes require a prescription for dispensing all medically important antimicrobials (MIAs) [10]. In the USA, effective January 2017, the Veterinary Feed Directive requires all medically important antimicrobials to be used in animal feed or water, to have veterinary oversight [11]. Further, in 2021 the United States Food and Drug Administration (FDA) published Guidance 263 in which drug manufacturers were asked to voluntarily bring MIA under prescription-only status from over-the-counter sales for existing and new antimicrobials [12]. Guidance 263 also requires remaining over-the-counter products to display a warning label limiting use to or by order of a veterinarian and requiring a prescription for purchase [12].

In both Canada and the U.S., federal authorities collaborate with national veterinary associations to support veterinarians and ultimately producers in antimicrobial stewardship, by monitoring data on resistance and antimicrobial use [13,14]. The two jurisdictions use slightly different definitions (e.g., for Medically Important Antimicrobials, or MIAs), metrics, and time intervals captured by the current reporting system (with the U.S. system capturing data from 2012 on, and the Canadian system from 2018). However, in both the USA and Canada in 2021, the most recent year for which antimicrobial sales are data for livestock are available, cattle were a major source of livestock AMU by weight for both medically important and non-important antimicrobials, and this intrinsically has public health implications as noted above [13,14].

The magnitude of AMU and AMR have not been specifically summarized for the relatively extensively managed North American cow–calf production sector, although differences in AMU and AMR between the cow–calf and more intensively managed feedlot sectors have been reported [15,16]. This lack of review of beef cow–calf AMU and AMR could have several reasons. The populations of interest (cows and pre-weaning calves) are logistically relatively difficult to sample while out on pasture. Beef cow–calf AMU data are not always available [17,18] and when present, recording methods may vary [19]. The lack of standardized records systems is an industry gap that will be challenging to mitigate given the wide variation in needs and interests of cow–calf producers. The cow–calf sector primarily consists of independent owners and businesses of varying sizes using a broad range of records systems, relative to the feedlot sector which is dominated by a much smaller number of larger production units, making sampling for AMR monitoring simpler. Antimicrobial use and AMR have been studied more frequently in feedlot cattle as AMU is typically greater under intensive management conditions and feedlot animals which are close to slaughter are of greater interest to public health. The regulatory framework for AMU and antimicrobial prescriptions by veterinarians have changed over the past decade in the USA and Canada [10,11], further suggesting the need for a review.

Scoping reviews are appropriate for assessing the extent of the knowledge in an emerging field, or to identify, map, report or discuss the characteristics/concepts in that field [20]. In contrast with the systematic review, which is used to study more precise research questions such as the effectiveness of a specific intervention, scoping reviews are used if the purpose of the review is to identify knowledge gaps, scope a body of literature, clarify concepts or to investigate research conducted [21].

A scoping review was deemed appropriate as the aim of this review was to describe the published research available to support risk-based policy-making for AMR and antimicrobial stewardship initiatives in the North American beef cow–calf sector. The objectives of this scoping review were to map AMU/AMR research conducted in the North American beef cow–calf sector, summarize published AMU/AMR literature, describe predictors for resistance development in the beef cow–calf sector, and identify research gaps pertaining to the development of AMR risk mitigation strategies. The review specifically targeted animals not identified as being sick to summarize AMR data from the baseline population and to avoid laboratory diagnostic samples from sick animals that had potentially been treated with antimicrobials multiple times.

## 2. Results

### 2.1. Overview of Captured Studies

**Figure 1:**
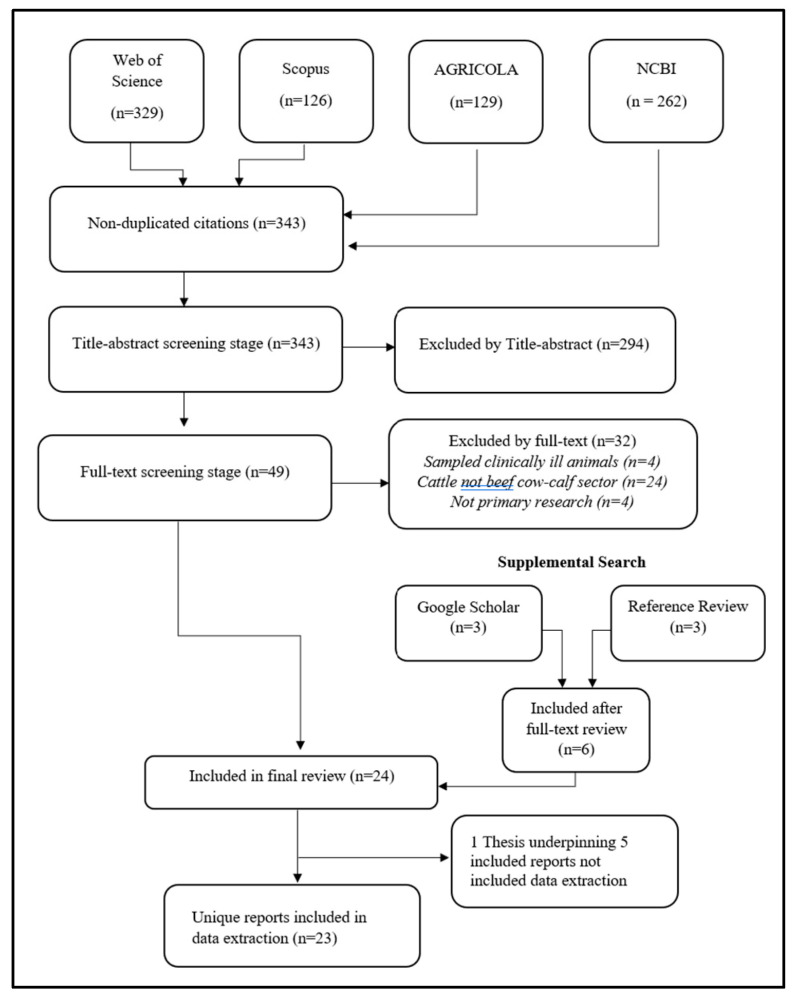
Flow of citations through review process to identify published research on antimicrobial use and antimicrobial resistance in beef cow–calf herds in the United States and Canada.

The electronic bibliographic search yielded 343 unique citations, including the additional citations captured in the follow-up search conducted in June 2023. First-level title and abstract screening excluded 294 publications, for reasons including only sampling out-of-scope populations (e.g., dairy cattle, feedlot) and sampling locations (e.g., Mexico or Europe). Another 32 publications were excluded after full-text review, for reasons including sampling only clinically ill animals, sampling animals not identifiable as beef cow–calf sector, and not reporting primary research. The Google Scholar search additionally identified one relevant thesis [22], one journal article, and one government report. Hand searches of relevant publication references yielded three additional potentially relevant citations. Twenty-four publications met the inclusion criteria, including one thesis which underpinned four other included publications; thus, twenty-three unique publications underwent data extraction. Of these, twenty-one were identified in the original search, none in the second search in June 2022, and two in the final search in June 2023. A diagram of the review process is presented in Figure 1.

Of the 23 relevant publications, 10 were conducted in the USA and 13 in Canada between 1999 and 2020. Thirteen described investigations of AMR, twelve studied AMU, and two studied both. The most frequently studied population was pre-weaned calves (n = 17), followed by cows (n = 14) with breeding bulls the least frequently described population (n = 2). Two AMU studies investigating producer attitudes studied usage at the herd level and did not report usage stratified by class of cattle [17,18]. The number of herds studied varied from one university research herd [23] to 2159 commercial herds participating in a USA federal survey [24]. Participant herd size varied across studies, with some investigators excluding smaller herds, as defined in the study, and some including them. A descriptive summary of the included studies is presented in Table 1.

**Figure 2:**
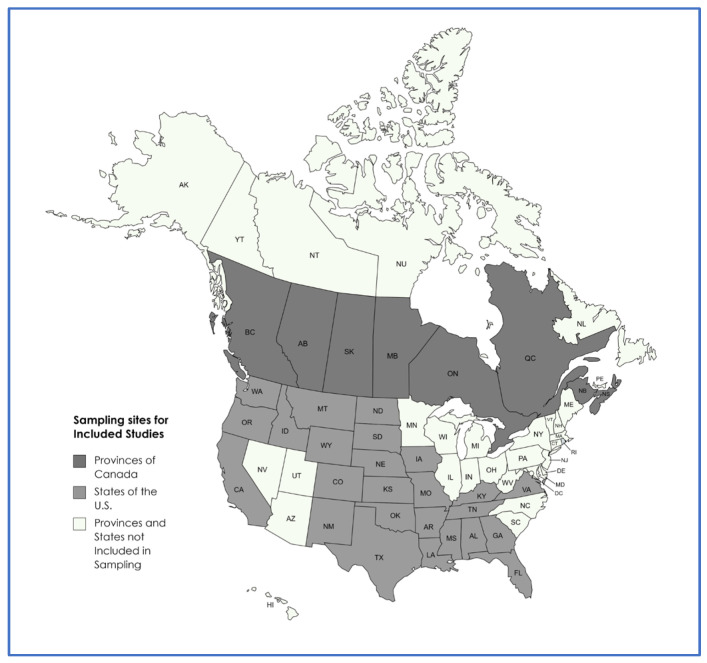
Sampling locations of included studies.

A map displaying the provinces and states from which samples originated, across the included studies, is presented in Figure 2.

### 2.2. Studies Reporting Antimicrobial Use

While several investigators have identified the value of studying individual animal data in verifying producer reporting of AMU [40,41], only two studies in this review captured individual animal data [16,37]. All twelve included AMU studies reported counts (e.g., number of farms studied reporting usage of antimicrobial ”x” over time period “y”) as an outcome metric, with one additionally reporting weight and average animal daily dosage [16]. Medically important antimicrobial use was specifically captured in most AMU studies (n = 7/12), as were potential AMU predictors (n = 10/12). A summary of the characteristics of the 12 included AMU studies is presented in Table 2.

Nine AMU studies described herd-level usage (Yes/No) for classes of antimicrobials (e.g., cephalosporins) or specific antimicrobials (e.g., ceftiofur), aggregated as counts of herds that reported use (Table S1). The panels of antimicrobials for which use was captured varied across studies, as did the age/class of cattle studied, with five describing use aggregated across all classes of cattle, four describing use in cows, and four describing use in calves. A summary of the reported prevalence of herd-level AMU choices for eight of the most frequently investigated classes or examples of antimicrobials is presented in Table S1. One set of studies in this dataset examined different aspects of AMU and AMR in the same study population [30,31,32]. Two studies from which quantitative estimates of use were extracted did not investigate prevalence by identifying the number of producers using a given antimicrobial but instead asked which antimicrobials were used most frequently [6,18].

The objectives for AMU were reported in seven of 12 included studies, ranging from “Prevention, control, or treatment” [6] to “Treatment or Prevention” [7,24,31,32] to “Treatment” [33,37]. These papers also identified the herd-level frequency of major disease syndromes requiring treatment, and in some studies, findings were presented by age category (Table 2).

Tetracycline use was reported by most herds in the identified studies, ranging from 36% [18] to 88% [16] for injectables (Table S1). Beta-lactam penams and florfenicol were the next most common antimicrobials reported for herd use with the relative frequency varying depending on whether the population was “all cattle”, cows, or calves. Reported herd-level macrolide use varied across cattle populations (cows vs. calves), and whether the broad group or specific generic macrolides were the captured outcome. Cephalosporin use was reported by 18% or less of herds across this group of studies. Reported antimicrobial use was greater in calves relative to cows for phenicols, diaminopyrimidines–sulfonamides, macrolides, cephalosporins, and fluoroquinolones (Table S1). Capture of administration routes varied across studies (Table S1).

Population-averaged individual-level reasons for AMU were reported by U. S. [6,24] and Canadian [37] investigators. All similarly reported lower individual-level use relative to herd-level, and relatively low frequency of AMU (e.g., <10% of calves treated for each of the major disease syndromes captured) overall. Major disease syndromes treated varied with the class of cattle studied, for both herd- and individual-level usage. Respiratory and digestive disease, and umbilical infections, were major reasons for disease treatment in pre-weaning calves, with pinkeye and lameness being major reasons for AMU in cows, across these studies.

Within-herd AMU was reported as categorical summaries from herd records in two Canadian studies [7,31] for each of the classes of livestock on-farm (e.g., cows, pre-weaning calves, post-weaning calves, and bulls). Overall, reported within-herd AMU was relatively low. In the study collecting data in 2013–2014, 71% of herds reported that less than 5% of cows in the herd received any antimicrobial, and 46% of herds reported that less than 5% of calves received any antimicrobial [31]. Corresponding reporting from the 2019–2020 study, indicated of 91% of herds reported treating less than 5% of cows, and 88% of herds reported treating less than 5% of calves with any antimicrobial [7]. While more herds reported treating calves with respiratory disease, the frequency of calves treated within affected herds was higher for neonatal diarrhea. Lameness was the most common reason for treating cows and bulls.

A broad range of potential predictors for herd-level reporting of AMU was investigated in the captured literature, and the variables investigated for association with AMU are presented in Table S2. Significant (*p* < 0. 05) predictors included herd size, with larger herds more likely to report AMU; and classes of livestock, with greater odds of AMU reported in calves relative to cows for multiple antimicrobials investigated (Table S2). Other associated factors included disinfection of water troughs, with herds reporting regular disinfection having reduced odds of AMU; quarantining of introduced livestock, with herds employing quarantine having reduced odds of AMU; and marketing strategies, with herds retaining ownership of calves post-weaning having increased odds of any Health Canada Category I drugs used in the herd (Table S2).

Verification of the questionnaires used for data collection in the AMU surveys captured was reported in two studies [16,37].

### 2.3. Studies Describing Antimicrobial Resistance

Thirteen studies investigated AMR in the North American beef cow–calf sector, with six conducted in the USA and seven in Canada (Table 3). Two studies [26,27] employed deep nasopharyngeal (DNP) swabs to capture the prevalence of potential respiratory pathogens including *Histophilus somni*, *Mannheimia haemolytica*, *Mycoplasma bovis*, and *Pasteurella multocida*. Sample matrices for the remaining studies were either feces or colon content, with *Escherichia coli* (*E. coli*) included in all but two of these studies and other enteric bacteria (*Campylobacter* spp., *Salmonella* spp., *Enterobacter* spp.) less frequently reported (Table 3). Two studies pooled samples, with the remainder (n = 11) using individual sampling (Table 3).

Broth microdilution was the most frequently used method of antimicrobial susceptibility testing (n = 9 studies, Table 3). Antimicrobial resistance in *E. coli* was the most frequently investigated bacterial species (n = 9), and all *E. coli* studies used breakpoints endorsed by the Clinical and Laboratory Standards Institute (CLSI) or its predecessor, the National Committee for Clinical Laboratory Standards (NCCLS). Other breakpoints (Antimicrobial Resistance Research Unit, Agricultural Research Service, United States Department of Agriculture; National Antimicrobial Resistance Monitoring System) were reported in three studies investigating *Campylobacter* spp. Only three studies reported *Salmonella*, and in one of the two studies, there was only one isolate.

Study outcomes were reported as counts in 12 of 13 AMR studies (Table 3), with 1 describing a “resistance index”, calculated by dividing the sum of codified resistance of all isolates by the total number of isolates [35]. The dataset for the AMR studies had a hierarchical structure; for example, isolates within individuals within herds within the industry sector (e.g., cow–calf) within studies. Antimicrobial resistance findings were aggregated and reported at various hierarchical levels among the captured studies. A summary of the characteristics of included AMR studies is presented in Table 3.

Predictors significantly (*p* < 0.05) associated with AMR in one or more studies included herd attributes such as calf mortality (with greater mortality positively associated with AMR) [30] and calf age in spring (<3 d relative to >10 d) [34], with a complete list outlined in Table S3. Calves were identified as having greater odds of AMR relative to cows when measuring sulfamethoxazole resistance [15]. An increase in the odds of tetracycline resistance in calves was reported with an increasing proportion of cows being positive for tetracycline resistance, with a similar relationship reported for sulfamethoxazole when sampling occurred in the spring [34]. One study reported a significant positive association between herd size and AMR [29]. The magnitude of statistical significance varied across specific outcome metrics (Table S3).

Several herd-level management practices, such as regularly disinfecting water troughs, and quarantining new arrivals to the herd, were associated with significantly reduced odds of AMR [29]. While many aspects of herd-level AMU were examined for a potential association with AMR (Table S3), only two were reported to be statistically (*p* < 0.05) significant. Ionophore feeding was reported to have a significant (*p* < 0.05) association with decreased odds of cefotaxime-resistant bacteria [29]. Herds in which cows were treated with florfenicol had increased odds of *E. coli* resistance to ≥2 antimicrobials [30].

Individual-level factors significantly associated with AMR included calf age, with calves greater than 10 days of age having increased odds of AMR relative to those less than three days old [34], and previous illness, with those classified as “unhealthy” having increased odds of AMR [36]. A complete list of potential AMR predictors reported in the captured literature is presented in Table S3.

The most frequently reported AMR outcome in *E. coli* bacteria was resistance to one or more antimicrobials, reported in five publications representing fifteen studies across various populations and hierarchical levels of aggregation. A summary of the most frequently reported quantitative AMR outcomes in enteric bacteria originating from North American beef cow–calf herds is presented in Table S4. One included study [29] reported AMR in environmental samples including forage, water, and soil.

The most frequently reported AMR outcomes were *E. coli* resistance in cows to one or more of the following antimicrobials: cephalosporins, tetracyclines, sulphonamides, or aminoglycosides. Resistance varied across antimicrobials, populations sampled, and level of aggregation. Resistance in *E. coli*, aggregated at the isolate level, was relatively low, with AMR occurring more frequently in samples originating from calves relative to cows (Table S4).

Resistance varied across classes of antimicrobial and specific examples selected, with generally greater frequency of resistance reported in tetracyclines and sulfonamides, relative to classes more important in human medicine such as cephalosporins (e.g., ceftiofur) or diamino-pyrimidines (e.g., trimethoprim) (Table S4). Findings were most frequently reported aggregated at the isolate level; however, in the small number (n = 2) of studies reporting AMR aggregated at more than one level, resistance was most frequent at the herd level and less frequent at the individual animal or isolate level for a given drug tested (Table S4).

## 3. Discussion

Overall, a relatively small number (n = 23) of relevant publicly available studies of AMU or AMR in North American beef cow–calf production were captured. These largely originated from several research or surveillance groups: the Western College of Veterinary Medicine (n = 10 studies), the United States Department of Agriculture (National Animal Health Monitoring System (NAHMS)) (n = 3), Ontario Veterinary College (n = 2), and the College of Veterinary Medicine of the University of Tennessee (n = 2). However, this coverage emphasizes the major beef-producing regions of the U.S. and Western Canada [6,7].

The most recent sampling captured is from 2019–2020, reported in only one Canadian study [7]. Significant changes to veterinary antimicrobial prescribing occurred relatively recently, in both the USA and Canada. Most studies included in this review were conducted prior to these important regulatory changes.

The value of using multiple information sources to validate AMU data, especially including individual treatment records, has been previously reported [41,42]. However, only two of the twelve included AMU studies used individual treatment records as the primary data source [16,37]. While a recent review [43] of AMU metrics proposed that investigators should justify their use of AMU metrics, this was rarely reported by the studies captured in this review. The two studies using multiple metrics reported the reasons for their choices; the remaining captured studies did not.

Most (n = 11) of the AMU studies in this review reported count-based AMU metrics only. This is especially noteworthy as one study that presented multiple usage metrics found comparative usage across groups varied based on the outcome metric selected [16]. This observation was also reported in the feedlot with investigators reporting that ranking of the antimicrobials by use to guide AMU mitigation strategies varied with the metric selected [41].

Studies reporting herd-level AMU alone do not fully capture herd exposure to AMU. Two studies capturing AMU choices for herds, as well as the proportion of individual animals treated within herds, reported a relatively small proportion of individuals being treated within herds reporting usage of a given antimicrobial [7,31]. Population-averaged estimates of individual use reported for major diseases requiring AMU similarly reported relatively low frequency of use relative to herd-level estimates [6,24,37]. This suggests that studies reporting only herd-level AMU lack important information required for estimating antimicrobial exposure. Additionally, herd-level investigations of AMU not quantifying overall AMU by drug or class, but only describing usage by major disease syndromes, also are missing an important parameter of AMU.

Investigation and reporting of beef cow–calf sector AMU are contingent on producers securing this information in a retrievable form, preferably with individual animal records. One study included in this review [31] reported that of 100 participants, 60 reported on record-keeping in a complementary 2015 survey, with 49 participants having health records for their cattle, suggesting potential information gaps with some herds [31]. Quality assurance programs such as Verified Beef Plus™ typically require health record-keeping, but the various formats allowed (hardcopy notebooks, electronic spreadsheets) could be challenging for third parties to collate for AMU studies [19]. The capture of herd records, particularly individual treatment records, could support survey verification, reported in only two of the included AMU studies [16,37], and help researchers mitigate potential survey recall bias.

Investigation of whether antimicrobials are being used prudently (“giving the right drug to the right animal at the right time”) similarly would require knowing the true diagnosis, the producer’s diagnosis, what the veterinarian prescribed for treatment, and whether/how accurately this was administered by the beef producer. Confirmation of producer diagnoses by a veterinarian is not practical or even possible in many instances due to the distance of cattle from the nearest clinic, as well as limited opportunities for observation, restraint, and timely treatment in extensively managed animals. Telemedicine opportunities are increasing, but cell phone coverage is inconsistent in many areas used for grazing. These questions also necessitate accurate individual treatment records, as well as substantial time and resources to correlate records to other information sources including herd protocols, drug invoices, and garbage can audits. Studies assessing prudent use are rarely reported, even in more intensively managed and therefore less logistically demanding sectors such as dairy and swine production, and no such investigation of prudent use in the beef cow–calf sector was captured in our search. When executed, these studies can be informative: a survey of Danish dairy farms validating treatment records with other information sources reported that of 12,930 beta-lactam drug use records, 1669 were under the manufacturer’s recommended daily dose and 6814 were over [42]. However, this kind of investigation of beef cow–calf operations remains impractical given the extensive nature of production.

Understanding the herd manager’s process for making treatment decisions is fundamental to assessing the prudent use of antimicrobials in beef cow–calf operations. This intrinsically involves documenting the use of treatment protocols drafted by the attending veterinarian. One Tennessee study of producer attitudes towards antimicrobial use reported that of 147 cow–calf producers responding to this question, 15% (22/147 (respondents) had written treatment protocols for the treatment of sick animals [17]. An earlier study conducted in Tennessee reported that Beef Quality Assurance or master beef producer certification, as well as the producer having written instructions for treating sick animals, were significantly associated with AMU, suggesting the importance of both producer quality assurance programs and the availability of written treatment protocols in supporting prudent antimicrobial use [18] (Table S2). Additional questions around prudent use include whether veterinarians attending beef cow–calf herds consistently provide clear, understandable treatment protocols, whether these provide algorithms to guide producers’ decision-making, and whether veterinarians are available ad hoc to offer advice as needed, to guide producers in this area. Studies specifically describing the activities of producers and veterinarians in the process of decision-making for AMU, reporting AMU or AMR as an outcome of interest were not captured by our search and are currently research gaps. In fact, the AMU data captured by our review originated exclusively from producers (as opposed to veterinarians). Similarly, a 2020 review of approaches for improving antimicrobial stewardship in livestock farmers and veterinarians did not capture any programs or interventions for producers, or “facilitators” implemented by veterinarians to improve producer prudent use, sampling the beef cow–calf sector [44].

While studies aiming to characterize antimicrobial exposure should ideally include both the level (antimicrobial agent, daily dose administered, and numbers of treated individuals) and duration of exposure, this information was reported in only one included study [16]. This study was limited to eight relatively small herds. This can be challenging information to collect for larger numbers of privately owned, and more extensively managed herds, and for antimicrobials with various durations of action (e.g., short- vs. long-acting injectables).

If the AMU data are to be used for benchmarking, comprehensiveness of the data and representativeness of study target populations are crucial. Antimicrobial use is intertwined with herd health and influenced by a range of management practices such as calving heifers and cows together, or failure to sort cow–calf pairs out of the calving area [45]. Reporting of these predictors was limited within many of the studies.

The chronological time of sampling is a potential predictor of interest to industry and policy-makers. However, given the relatively small number of studies included in this review, the range of outcomes and other potential predictors (e.g., pre-weaning calves vs. cows) [31], expected differences based on seasons of the year [34], and level of data aggregation (such as individual vs. herd) [31] reported, potential changes in AMU over time would be difficult to assess from the current dataset.

Comparability is an important consideration across study populations, sub-populations (e.g., pre-weaned calves), or herds. The population at risk can vary by population size and structure, average weight at treatment, as well as the risk period for being treated [43]. Within the included studies, descriptions of AMU were presented stratified by some of the major classes of livestock in a beef cow–calf operation (e.g., breeding cows, pre-weaning calves) in eight studies, supporting the identification of groups with greater AMU (Table 2). Despite the strongly seasonal nature of production activities and attendant management procedures in cow–calf herds, only one captured study stratified AMU by season [34]. The scarcity of data pertaining to this element of context is an important research gap.

Several differences were noted between Canadian and USA AMR studies. The Canadian *E. coli* AMR studies tended to test for a group of antimicrobials similar to the Canadian Integrated Program Antimicrobial Resistance Surveillance (CIPARS) panel [15], using CLSI-endorsed breakpoints, while the USA *E. coli* AMR studies used NARMS breakpoints. The 2008 USA NAHMS studies [24] represents the largest sample of North American cow–calf herds within the review dataset of captured studies.

*Escherichia coli* was the most frequently reported enteric bacterial target, possibly due to its value as an indicator organism with potential public health impact [46]. Selection of *E. coli* precludes the effective assessment of the samples for resistance to many macrolides important in veterinary medicine due to intrinsic resistance [47], leaving a knowledge gap for the frequency of resistance to a group of antimicrobials reported as used relatively frequently in the captured studies (Table S4).

Cows were sampled more frequently than calves, likely reflecting the relative ease of handling for both groups. The outcomes measured were reported at levels ranging from isolate to industry sector (e.g., cow–calf vs. feedlot), and this contributed to a relatively small group of surveys per outcome which could be compared or potentially pooled for summary estimates.

The twelve captured studies of AMR fell into two broad groups: those studying respiratory pathogens such as *Mannheimia haemolytica* (n = 2) and those studying enteric organisms (n = 10). The two studies of respiratory pathogens did not report risk factors occurring pre-weaning for AMR, given the studies’ focus on AMR trends throughout the production cycle. In combination with the relatively small number of herds sampled (23 in total), research gaps remain in improving our understanding of AMR prevalence and risk factors for important pathogens. The finding of “unhealthy” calves having increased odds of AMR suggests the possible importance of some herd health and herd-level management procedures in mitigating AMU [36]. A recently published study of 18 cow–calf farms (which included organic farms) reported that herd information (type of ranch and age of animals) and nutrition-related factors (cleaning of water troughs, use of bleach by farmers for water trough cleaning, and provision of mineral supplement to calves) accounted for approximately 52% of the total variance in the data [48].

Multiple potential sources of bias exist within and across the included studies, including selection bias at both the herd and individual levels of sampling. Recruitment of herds to observational studies requires owner consent, and this inherently introduces selection bias for owners having the interest and willingness to contribute time and resources to allow physical sample or data collection [43]. Individual sample collection for the AMR studies included convenience sampling (n = 5 studies), with the direction of bias difficult to predict. Several different components of potential information bias exist, with the likely direction of bias varying across components. A relatively small proportion (2/12) of AMU studies reported the use of additional data sources for verification of reported AMU, to minimize reporting bias [16,37]. Additionally, some herd records were noted to be incomplete in both of the studies employing herd records as data sources [16,37]. One Canadian study [49] reported that study herds declining participation were less likely to have complete calving records suggesting that herds recruited in the included studies might not be representative of the North American herd overall. The likely direction of this bias is difficult to predict given the scarcity of reporting on beef cow–calf herd AMU record quality.

For some time, technological advances have been suggested as potential opportunities to reduce AMU or mitigate AMR [50]. Within the captured studies, several management practices were identified that were significantly associated with reduced AMU (e.g., use of calf diarrhea vaccines, early in the year vs. later start to calving season, biosecurity practices, Table S2) and AMR (e.g., biosecurity practices such as cleaning water troughs, Table S3). No specific technological advancements associated with reduced AMU or AMR were captured in our dataset. However, several broad areas of current investigation were identified, although not included in data extraction as they do not directly report an outcome of interest. For example, the use of non-pathogenic bacteria to beneficially modulate the animal’s microbiota was reported by multiple groups [51,52]. Other areas of investigation, such as ancillary treatment for calf diarrhea to minimize antimicrobial use [53], therapeutic use of colostrum in diarrheic calves [54], and breed differences in susceptibility to AMR [55] were also reported. Additional studies of management or technological strategies specifically reporting impacts on AMU or AMR as a measured outcome would be very useful in providing evidence required for risk assessment to support risk-based policy-making.

Given the complexity of the dataset, with a myriad of potential herd- and individual-level predictors, and the identified data gaps, the studies captured in this review collectively provide incomplete evidence required by policy-makers to support benchmarking or to inform risk models in support of public health. For example, when reporting AMU by drug class, an accompanying listing of the specific drugs captured by the study survey would be helpful in supporting the assessment of the comparability of findings across studies. More detailed reporting of drugs captured by each survey would also support differentiating the use of “long-acting” vs. repeatedly dosed products, which is of interest pertaining to the logistics underlying drug choices, and also the potential for longer exposure to a lower level of the active drug. However, an issue in extensively managed operations is that short-acting drugs might only be used once due to difficulty in capturing the animal a second or third time—leading to incomplete treatment and increased risk of AMR. Single-dose drugs could logistically be preferable at times, especially in animals on pasture, in that single-use drugs could be more effective in clearing the original infection if used in the right animals at the right time.

Producers and veterinarians additionally need to continue to improve documentation and tools to promote responsible management of AMU in the beef cow–calf sector, to inform the social license to operate, and to prevent potential treatment failure associated with AMR. This requires identification of the major purposes for AMU, and those diseases most frequently associated with the use of drug classes important in human medicine. In Canada, MIAs are categorized as Very High Importance, High Importance, or Medium Importance based on indications for usage in human medicine and available alternatives [10]. In the USA, MIAs are categorized as Critically Important, Highly Important, or Important, based on five criteria [12].

Current findings across this dataset suggest a relatively small proportion of cattle receive antimicrobials in the cow–calf sector. There is, however, evidence to suggest areas for potential improvement. Beef calves in spring are the most likely group on-farm to receive treatment [36] including some drugs categorized as of “High Importance” in human medicine [10]. Therefore, producers and veterinarians should work together to address knowledge gaps such as the frequency and reasons for usage of drugs for individual animals, particularly macrolides, in pre-weaning calves, and whether management practices can reduce usage. Given that most AMR studies focus on *E. coli* as the microbial target of interest, additional studies of AMR with other enteric organisms as well as for respiratory pathogens in pre-weaning calves would be useful in prioritizing disease syndromes for AMU mitigation strategies. Pre-weaning calves are of particular interest to the industry as they represent the risk of AMR in calves entering the feedlot, potentially limiting options for treating bovine respiratory disease.

One included study describes the isolation of AMR bacteria from the environment of cow–calf operations [29], as do other published studies sampling farms with minimal or no antimicrobial use [48,56]. One investigation additionally reported finding no significant association between herd management practices including AMU, and AMR in *E. coli* isolates from cow–calf operations suggest the importance of further investigating other potential drivers of AMR such as the environmental burden of AMR in cow–calf production [48].

This scoping review had several potential limitations. We did not chart data specifically pertaining to risk of bias (ROB) per se given the current lack of widely endorsed guidelines for assessing individual observational studies for ROB or incorporating ROB in furthermore focused syntheses such as systematic review and meta-analysis of observational studies [57,58]. We did however capture major potential predictors of AMU and AMR such as the age of cattle, where this information was reported. While genomic-level study of antimicrobial resistance was reported in a relatively small number of captured studies [23,25,26,27,38] charting genomic AMR data was not an objective of this work, and we did not include genomics terms in our search strategy. Additional scoping reviews targeting and charting the available data from the cow–calf sector could provide additional insight into potential antecedents of AMR in the feedlot sector, closer to consumers in the farm-to-fork continuum.

## 4. Materials and Methods

This scoping review was conducted following a protocol informed by Joanna Briggs Institute (JBI) Evidence Synthesis [20] and the Preferred Reporting Items for Systematic Reviews with Extension for Scoping Reviews [59]. A copy of the study protocol and the preferred reporting items checklist is available online as Supplementary File S1.

### 4.1. Eligibility Criteria and Search Strategy

Given the aim of the review, the condition, context, and population (CoCoPop acronym) was used to characterize eligibility criteria [60]. The criteria are outlined as follows:

*Condition*: Antimicrobial use or antimicrobial resistance, or risk factors potentially associated with either of these, reported in any metric.

*Context*: Environmental factors can have an impact on the frequency of occurrence of the conditions of interest [60], and these factors (e.g., geographic location and season), were included when reported by authors.

*Population*: Studies with healthy/asymptomatic animals produced in beef cow–calf herds in Canada and the USA were eligible. Studies of all classes of livestock within the cow–calf sector (pre-weaning calves, breeding heifers and cows, breeding bulls) were eligible. For studies reporting data from cow–calf operations as well as other enterprises such as stockers, backgrounding or feedlots, or studies sampling operations which combined one or more of these enterprises with cow–calf production, sampling from the populations of interest was deemed relevant, and sampling originating from populations outside of scope (weaned calves or other feeder animals) was not included in data extraction.

*Inclusion:* Primary research investigating beef cow–calf populations in Canada and the USA, of any size, was eligible with no restrictions on year or language of publication. If the research included AMR outcomes, only data originating from sampling clinically healthy as opposed to ill animals were deemed relevant. Journal articles, government or industry publications, and theses were all deemed relevant.

*Exclusion*: Studies conducted in countries other than Canada and the USA were excluded to restrict data to regionally relevant herd management practices, licensed antimicrobials, and regulations limiting access. Additionally, AMR studies were excluded if they only sampled clinically ill or symptomatic animals, or they did not report outcomes identifiable as originating from clinically healthy animals or collected as part of routine surveillance from privately owned herds. Calf rearing operations, in which dairy calves not destined for the breeding herd are fed to slaughter, were outside scope, even if reporting on calves of beef breeds. Studies investigating cattle in feedlots or only, or only sampling cattle raised within organic or antibiotic-free production systems, or herds purposively selected for known minimal antimicrobial use, were also deemed outside of scope.

### 4.2. Search

An initial search was conducted on 22 January 2022 in three electronic bibliographic databases: Web of Science, Scopus, and AGRICOLA, using a three-term search. One term described the populations of interest (e.g., cows, calves, or beef cattle). The second term described the outcome of interest (e.g., AMR or AMU). The third term described location (e.g., Canada or USA). Examples of the specific search strategies employed in each database are presented in the study protocol in Supplementary File S1. A search in Google Scholar was also conducted using the same strategy as employed in the electronic bibliographic databases. Finally, a hand-search was conducted of the references section of relevant research studies and reviews to identify papers not captured by the electronic search. A follow-up search of all original sources was conducted on 20 June 2022. A final search was conducted 15 June 2023, which included all original sources as well as the National Center for Biotechnology Information (NCBI) Pubmed database.

### 4.3. Review Process, Data Cleaning and Analysis

A commercial spreadsheet program was used to manage the data (Microsoft Excel version 2304, Microsoft, Redmond, WA, USA). First-level relevance screening was performed independently by two reviewers on abstracts captured by the search. The first level screening form asked four questions: “Is the literature in the form of a journal article, thesis, or government report?”; “Does the research take place in Canada or the United States?”; “Does the study include data from cow-calf herds?”; and “Does the article contain information on AMU or AMR?”. Those citations deemed relevant using this form were promoted to second-level relevance screening, and the full paper was obtained.

The objective of second-level relevance screening was a full-text review by two independent reviewers to confirm eligibility. Relevant publications contained information on AMU or AMR in the cow–calf sector of the beef industry, specifically the Canadian or American cow–calf industries. First- and second-level relevance screening conflicts were resolved through discussion and confirmation by the senior author.

Third-level data extraction was performed by a single reviewer using a pre-tested data extraction form capturing major characteristics of each relevant study, such as year of sampling, populations studied, broad outcome(s) reported (e.g., AMU or AMR) and metrics used, as well as level of data aggregation (isolate/individual/herd). Copies of the three relevance screening forms used are available online in Supplementary File S1.

For purposes of this review, a study was defined as an investigation of AMU or AMR in a defined population, identifiable by beef cow–calf sub-populations (breeding cows, replacement heifers, pre-weaned calves, post-weaned calves, bulls, or all animals in the cow–calf herd). A publication (synonymous with “paper”) was defined as a publicly available description of one or more studies. The same study population could be sampled for different studies (e.g., the same group of herds could be the study population for one study describing AMU, and another describing AMR). Multiple studies could originate from the same overall study population, within one publication (e.g., AMU of a study group of herds could describe breeding cows and calves). For purposes of mapping the literature, all relevant publications were included in data extraction. If multiple publications sampled the same population for different studies and reported different outcomes, these were indicated with a superscript in tables.

Descriptive statistics were computed in a commercial spreadsheet program (Microsoft Excel version 2304, Microsoft, Redmond, WA, USA).

## 5. Conclusions

The dataset captured focused predominately on one AMU metric (count), studied at one hierarchical level (herd), and one population (cows) was investigated for AMR, leaving several major information gaps in most of the published literature. A small number of risk factors significantly associated with AMU (herd size, vaccine use, date of start of calving season) and AMR (biosecurity procedures) were reported. Additional studies targeting major reasons for AMU in calves, the impact of management procedures on usage, potential environmental sources of AMR, and AMR in respiratory pathogens and enteric organisms in addition to *E. coli* for pre-weaning calves are required to inform the development of AMR risk mitigation strategies. Studies specifically describing the decision-making processes of producers in AMU, which report AMU or AMR as an outcome of interest, are currently research gaps.

This research is especially important given the importance of AMU in cattle relative to overall livestock use in Canada and the USA.

## Figures and Tables

**Table 1:** Descriptive summary of characteristics of included studies of antimicrobial use and antimicrobial resistance in beef cow–calf herds in the United States and Canada.

Citation	Year of Sampling	Location of Sampling	Broad Topic	Study Population ^a^	Number Cow–Calf Herds	Herd Size Median (Range)
Fossen et al., 2023 [7]	2019–2020 ^b^	AB, BC, MB, ON, QC, NB, NS, SK, Canada	AMU ^c^	Cows, Calves, Bulls	146	Med = 124 Range (14–1044)
Schmidt et al., 2020 [25]	2018	FL, USA	AMR ^c^	Cows	N/R	N/R
Ekakoro et al., 2019 [17]	2018 ^b^	TN, USA	AMU ^c^	All	231	Categorized Min = 1–49 Max = 500+
Nobrega et al., 2021 [26]	2017	AB, Canada	AMR ^c^	Calves	22	Range (200, 3200)
Guo et al., 2020 [27]	N/R	AB, Canada	AMR ^c^	Calves	3	N/R
United States Department of Agriculture (USDA) et al., 2020 [28]	2017 ^b^	24 states ^d^ USA	AMU	Cows, Calves, Heifers	2013	Categorized Min = 1–49 Max = 200+
USDA et al., 2020 [6]	2017 ^b^	24 states ^d^ USA	AMU	Cows, Calves, Heifers	2013	Categorized Min = 1–49 Max = 200+
Markland et al., 2019 [29]	2016 ^b^	FL, USA	AMR ^c^	Cows, Calves	17	N/R
Waldner et al., 2019 [30]	2013–2014	AB, SK, MB, Canada	AMU, AMR ^c^	Cows	AMU = 98 AMR = 107	Med = 228 IQR (159–354)
Waldner et al., 2019 [31]	2013–2014	AB, SK, MB, Canada	AMU ^c^	Cows, Heifers, Calves, Bulls	100	Med = 234 IQR = 160–359
Waldner et al., 2019 [32]	2013–2014 ^b^	AB, SK, MB, Canada	AMU	Cows, Calves, All	100	Categorized Moderate = 100–300 head Large = 300+
Agga et al., 2016 [23]	2014	NE, USA	AMR ^c^	Cows	1	6000
Waldner et al., 2013 [33]	2010	BC, AB, SK, Canada	AMU	Calves	304	Med = 132 (5th, 95th%: 32, 529)
USDA et al., 2012 [24]	2008 ^b^	24 states ^d^ USA	AMU ^c^, AMR ^c^	Cows, Calves, Heifers	AMU = 2159 AMR = 173	Categorized Min = 1 head
Green et al., 2010 [18]	2007–2008	TN, USA	AMU ^c^	All	1024	N/R
Gow, 2007 [22] ^e^	2002–2003 ^b^	BC, AB, SK, Canada	AMU ^c^, AMR ^c^	Cows, Calves, Heifers	203	Med = 154 (53, 481)
Gow et al., 2008 [34] ^e^	2002–2003 ^b^	BC, AB, SK, Canada	AMR ^c^	Cows, Calves	2002 = 69 2003 = 10	2002: Med = 154 (74, 437) 2003: Med = 130 (86, 382)
Bae et al., 2005 [35]	2002–2003	WA, USA	AMR ^c^	Cows, Calves	3	N/R
Gow et al., 2008 [36] ^e^	2002 ^b^	BC, AB, SK, Canada	AMR ^c^	Calves	Spring = 91 Fall = 45	N/R; Herds < 50 excluded
Gow and Waldner, 2009 [37] ^e^	2002 ^b^	BC, AB, SK, Canada	AMU ^c^	Cows, Calves, Heifers	203	Med = 154 (53, 481)
Gow and Waldner, 2009 [38] ^e^	2002	SK, Canada	AMR ^c^	Calves	N/A Random isolates from [28,30]	
Berge et al., 2010 [39]	2001–2004 ^b^	CA, OR, WA, USA	AMR ^c^	Cows, Calves	9	N/R
Carson et al., 2008 [15]	2001 ^b^	ON, Canada	AMR ^c^	Cows, Calves	8	Med = 83 (9, 170)
Carson et al., 2008 [16]	1999–2002 ^b^	ON, Canada	AMU ^c^	Cows, Calves	13	Cow–calf: Med = 83 (9, 170)

^a^ Populations refer to those extracted from respective studies, within the scope of this review. Other column data fields, such as herd number and sampling, similarly refer to populations relevant to this review. ^b^ Representativeness of study population to target population expressly described by authors or included in discussion of selection bias.^c^ Primary study objectives. ^d^ Alabama, Arkansas, California, Colorado, Florida, Georgia, Idaho, Iowa, Kansas, Kentucky, Louisiana, Mississippi, Missouri, Montana, Nebraska, New Mexico, North Dakota, Oklahoma, Oregon, South Dakota, Tennessee, Texas, Virginia, and Wyoming. ^e^ These peer-reviewed publications describe individual chapters from a PhD dissertation [22]. Abbreviations: AMR = Antimicrobial Resistance; AMU = Antimicrobial usage; AB = Alberta; BC = British Columbia; MB = Manitoba; ON = Ontario; NB = New Brunswick; NS = Nova Scotia; QC = Québec; SK = Saskatchewan; TN = Tennessee; FL = Florida; NE = Nebraska; WA = Washington; USA= United States of America; N/A = Not applicable; N/R = not reported; Min = minimum; Max = maximum; Med = median; IQR = interquartile range.

**Table 2:** Descriptive summary of methods of 12 included studies of antimicrobial use in beef cow–calf herds in the United States and Canada.

Citation	Population ^a^	Sampling Year	Methods of Data Capture (Questionnaire Validation Reported)	Reason for Usage ^b^	Route of Usage	Time Interval Covered	Metric Used ^c^	Dosages Reported	MIA ^d^ Identified	Risk Factors Studied
Fossen et al., 2023 [7]	Cows, Calves, Bulls	2019–2020	Questionnaire (No)	Treatment, Prevention	Some	1 Year	Count ^e,f^	No	Yes	Yes
Ekakoro et al., 2019 [17]	All cow–calf	2018	Questionnaire (No)	N/R	No	N/R	Count ^e^	No	No	Yes
USDA et al., 2020 [6]	Cows, Calves, Heifers	2017	Questionnaire (No)	Prevention, Control or Treatment	Yes	1 Year	Count ^e,g^	No	Yes	Yes
USDA et al., 2020 [28]	Cows, Calves, Heifers	2017	Questionnaire (No)	N/R	Yes	1 Year	Count ^e^	No	No	Yes
Waldner et al., 2019 [30] ^h^	Cow, Calves	2013–2014	Questionnaire (No)	N/R	Some	1 Year	Count ^e^	No	Yes	No
Waldner et al., 2019 [31] ^h^	Cows, Calves, Bulls	2013–2014	Questionnaire (No)	Treatment, Prevention	Yes	1 Year	Count ^e^	No	Yes	Yes
Waldner et al., 2019 [32] ^h^	All cow–calf	2013–2014	Questionnaire (No)	Treatment, Prevention	Yes	1 Year	Count ^e,f^	No	Yes	Yes
Waldner et al., 2013 [33]	Calves	2010	Questionnaire (No)	Treatment	Yes	5 mo	Count ^e^	No	No	Yes
USDA et al., 2012 [24]	Cows, Calves, Heifers	2008	Questionnaire (No)	Treatment, Prevention	Yes	N/R	Count ^e,g^	No	No	Yes
Green et al., 2010 [18]	All cow–calf	2007–2008	Questionnaire (No)	N/R	Yes	N/R	Count ^e^	No	Yes	Yes
Gow and Waldner, 2009 [37]	Cows, Calves, Heifers	2002	Questionnaire (Yes), Diary, Individual Records	Treatment	Yes	6 mo	Count ^e,g^	No	No	Yes
Carson et al., 2008 [16]	Cows, Calves	1999–2002	Questionnaire (Yes), Diary, Garbage can audit	N/R	Yes	1 Year	Count ^e^, Weight ^i^ ADD	Yes	Yes	No

^a^ Populations refer to those extracted from respective studies, within the scope of this review. Other column data fields, such as herd number and sampling, similarly refer to study populations relevant to this review. ^b^ Studies reporting treatment as reason for usage also reported frequency of usage for major disease syndromes (e.g., respiratory disease) ^c^ Definitions as described in the scoping review protocol (File S1), i.e., count-, weight-, or dose-based. ^d^ MIA = medically important antimicrobials as defined by respective competent authorities. ^e^ Study described count of herds reporting usage. ^f^ Study reported within-herd usage. ^g^ Study reported population-averaged individual-level reasons for usage. ^h^ This study investigated a group of western Canadian beef herds recruited for a surveillance network targeting Alberta, Saskatchewan, and Manitoba, with various aspects of survey data reported in each superscripted citation. ^i^ Study used 2 weight-based measures: Weight = kg active ingredient per farm; Rate = mean kg of active ingredient disposed per 1000 animal-days. Abbreviations: N/R = not reported; ADD = average daily dose; Mo = month.

**Table 3:** Descriptive summary of methods of 13 included studies of antimicrobial resistance in beef cow–calf herds in the United States and Canada.

Citation	Population ^a^	Sample Matrix	Bacterial Species Studied	Sampling Year	Sampling Plan: Number (Detail) (Sampling Scheme)	Hierarchical Level Outcome Reporting	Outcome Metric Used ^b^	Method of Antimic-Robial Susceptibility Testing	Break-Points Used	Risk Factors Studied	Individual or Pooled Physical Sampling
**Non-Speciated Bacteria**											
Markland et al., 2019 [29]	Cows, Calves	Feces	Cefotaxime-resistant bacteria	2016	Herds: 17 (Random) Samples: 383 Calves, 457 Cows (Convenience) Environmental samples (n = 258)	Livestock Class	Count concentration	Broth microdilution	CLSI	Yes	Ind
**Enteric bacteria**											
Schmidt et al., 2020 [25]	Cows	Colon content	*E. coli*, *Salmonella* spp.	2018	Herds: N/R Samples: 535 cow carcasses. (Convenience) Herds: 17 (Random)	Sector	Count	Agar with antimicrobial	N/R	Yes	Ind
Waldner et al., 2019 [30]	Cows	Feces	*E. coli*, *Campylobacter* spp., *Salmonella* spp.	2014	Herds: 107 (Convenience) Samples: 107 (20 cows per herd pooled) (Systematic)	Isolate, Herd	Count	Broth microdilution	CLSI, NARMS	Yes	Pooled
Agga et al., 2016 [23]	Cows	Feces	*E. coli*, *Enterococcus* spp.	2014	Herds: 1 (Purposive) Samples: 369 (Cows ≥ 8 yr, receiving either ceftiofur or no AM tx) (N/R)	Treatment group	Count MIC distribution	Agar with antimicrobial	NARMS	Yes	Ind
USDA et al., 2012 [24]	Cows	Feces	*E. coli*, *Salmonella* spp., *Campylobacter* spp., *Clostridium difficile*	2007–2008	Herds: 175 (Convenience) Samples: N/R (up to 40 cows sampled depending on herd size) (Convenience)	Isolate	Count	Broth microdilution	CLSI, NARMS	Yes	Ind
Gow et al., 2008 [34]	Cows, Calves	Feces	*E. coli*	2002–2003	Herds: 2002 = 69 2003 = 10 (Convenience) Samples: 2002 = 533 Ind 2003 = 105 (Convenience)	Isolate, Ind, Herd	Count MIC distribution	Broth microdilution	CLSI	Yes	Ind
Bae et al., 2005 [35]	Cows, Calves	Feces	*Campylobacter jejuni*, *Campylobacter coli*	2002–2003	Herds: 3 (Convenience) Samples: 120 Ind (Convenience) (calves 2 to 4 weeks old (n = 20) and adult recently fresh cows (n = 20) per herd	Herd	Resistance Index	Agar dilution	ARR	Yes	Ind
Gow et al., 2008 [36]	Calves	Feces	*E. coli*	2002	Herds: Spring = 91 Fall = 45 Samples: Spring = 480 Ind Fall = 394 Ind (Spring: Convenience; Fall: Random)	Isolate	Count ^c^ MIC distribution	Broth microdilution	CLSI	Yes	Ind
Gow and Waldner, 2009 [38]	Calves	Feces	*E. coli*	2002	Herds: 57 Ind: 106 [Random)	Isolate	Count	Broth microdilution	CLSI	Yes	Ind
Berge et al., 2010 [39]	Cows, Calves	Feces	*E. coli*	2001–2004	Herds: 9 ≤ 22 calves that were 2 to 4 weeks old and10 cows that had recently calved.	Production type	Count	Agar dilution	CLSI	Yes	Ind
Carson et al., 2008 [15]	Cows, Calves	Feces	*E. coli*	2001	Herds: 13 Samples Pooled: N/R (pooled fecal pats collected 3 times per farm, 3 to 4 mo apart. Five groups of animals were selected)	Pooled: isolate, production type	Count	Broth microdilution	CLSI	Yes	Pooled
**Respiratory bacteria**											
Nobrega et al., 2021 [26]	Calves	DNP swab	*Histophilus somni*, *Mannheimia haemolytica*, *Mycoplasma bovis*, *and Pasteurella multocida*	2017	Herds: 22 (Convenience) Samples: 660 calves (30 calves per herd) (Random)	Isolate	Count	Broth microdilution	CLSI	No	Ind
Guo et al., 2020 [27]	Calves	DNP swab	*Histophilus somni*, *Mannheimia haemolytica*, *and Pasteurella multocida*	N/R	Herds: 3 (Convenience) Samples: 120 (40 calves sampled per participating herd) (N/R)	Isolate	Count	Broth microdilution	CLSI	No	Ind

^a^ Populations refer to those extracted from respective studies, within the scope of this review. Other column data fields, such as herd number and sampling, similarly refer to populations relevant to this review. ^b^ Outcome: Count = numbers or proportions; MIC distributions = tabular presentation of isolate minimal inhibitory concentrations, usually categorized, and including breakpoints for designating susceptible–intermediate–resistant; Resistance Index = the sum of codified resistance of total isolates divided by the total number of isolates. ^c^ Repeated measures of the study herds (cows or pre-weaned calves) reported, in a beef cow–calf population. Abbreviations: AM = antimicrobial; ARR = Antimicrobial Resistance Research Unit, Agricultural Research Service, United States Department of Agriculture; DNP swab= deep nasopharyngeal swab; CLSI = Clinical and Laboratory Standards Institute; NARMS = National Antimicrobial Resistance Monitoring System; *E. coli* = *Escherichia coli*; Ind = individual; Tx = treatment; Yr = year; Mo = month; MIC = minimum inhibitory concentration; N/R = not reported.

## Data Availability

None of the data were deposited in an official repository. The accompanying supplementary files provide the review protocol and the list of publications included at the data extraction level. Extracted data are included in the accompanying tables.

## Supplementary Materials

The following supporting information can be downloaded at: https://www.mdpi.com/article/10.3390/antibiotics12071177/s1, File S1. Protocol: Scoping review of antimicrobial use and antimicrobial resistance in the North American cow–calf sector, Table S1. Descriptive summary of selected herd-level outcomes of 9 included antimicrobial use studies in beef cow–calf herds in the United States and Canada, Table S2. Risk factors for antimicrobial usage reported in 7 included studies in beef cow–calf herds in the United States and Canada, Table S3. Risk factors associated with antimicrobial resistance reported in 7 included studies in beef cow–calf herds in the United States and Canada, Table S4. Descriptive summary of antimicrobial resistance of enteric bacteria reported in 6 included studies of antimicrobial resistance in beef cow–calf herds in the United States and Canada.

## References

[1] Strategic Framework for Collaboration on Antimicrobial Resistance. https://www.who.int/publications/i/item/9789240045408.

[2] Newman L., Tewari R., Darroch B. (2020). Consumer perception of antibiotic-free and hormone-free meat products. J. Food Stud..

[3] Working Principles for Risk Analysis for Food Safety for Application by Governments. https://www.fao.org/documents/card/en/c/fdaaa09d-8a3f-50c6-b801-945ffcac73a2/.

[4] Terrestrial Animal Health Code Volume 1. https://www.woah.org/en/what-we-do/standards/codes-and-manuals/terrestrial-code-online-access/.

[5] Pires S.D.M., Duarte R., Sofia A., Hald T. (2019). Source attribution and risk assessment of antimicrobial resistance. Microbiol. Spect..

[6] Beef Cow-Calf Health and Management Practices in the United States, 2017, Report 2. https://www.aphis.usda.gov/animal_health/nahms/beefcowcalf/downloads/beef2017/beef-2017-part2.pdf.

[7] Fossen J.D., Campbell J.R., Gow S.P., Erickson N., Waldner C.L. (2023). Antimicrobial use in Canadian cow–calf herds. Vet. Sci..

[8] Antimicrobial Stewardship Definition and Core Principles 2023. https://www.avma.org/resources-tools/avma-policies/antimicrobial-stewardship-definition-and-core-principles.

[9] SAVI: The Stewardship of Antimicrobials by Veterinarians Initiative 2023. http://savi.canadianveterinarians.net/en/home/.

[10] Responsible Use of Medically Important Antimicrobials in Animals. https://www.canada.ca/en/public-health/services/antibiotic-antimicrobial-resistance/animals/actions/responsible-use-antimicrobials.html.

[11] Veterinary Feed Directive (VFD) Basics 2022. https://www.avma.org/resources-tools/one-health/antimicrobial-use-and-antimicrobial-resistance/veterinary-feed-directive-basics.

[12] Guidance for Industry #263: Recommendations for Sponsors of Medically Important Antimicrobial Drugs Approved for Use in Animals to Voluntarily Bring under Veterinary Oversight All Products that Continue to be Available Over-the-Counter. https://www.fda.gov/media/130610/download.

[13] 2021 Veterinary Antimicrobial Sales Highlights Report 2022. https://www.canada.ca/en/health-canada/services/publications/drugs-health-products/2021-veterinary-antimicrobial-sales-highlights-report.html.

[14] 2021 Summary Report on Antimicrobials Sold or Distributed for Use in Food-Producing Animals. https://www.fda.gov/media/163739/download.

[15] Carson C.A., Reid-Smith R., Irwin R.J., Martin W.S., McEwen S.A. (2008). Antimicrobial resistance in generic fecal *Escherichia coli* from 29 beef farms in Ontario. Can. J. Vet. Res..

[16] Carson C.A., Reid-Smith R., Irwin R.J., Martin W.S., McEwen S.A. (2008). Antimicrobial use on 24 beef farms in Ontario. Can. J. Vet. Res..

[17] Ekakoro J.E., Caldwell M., Strand E.B., Strickland L., Okafor C.C. (2019). A survey of antimicrobial use practices of Tennessee beef producers. BMC Vet. Res..

[18] Green A.L., Carpenter L.R., Edmisson D.E., Lane C.D., Welborn M.G., Hopkins F.M., Bemis D.A., Dunn J.R. (2010). Producer attitudes and practices related to antimicrobial use in beef cattle in Tennessee. J. Am. Vet. Med. Assoc..

[19] Must Do Requirements—Summary. http://www.verifiedbeef.ca/producer-resources/must-do-requirements.cfm.

[20] Peters M.D.J., Marnie C., Tricco A.C., Pollock D., Munn Z., Alexander A., McInerney P., Godfrey C.M., Khalil H. (2020). Updated methodological guidance for the conduct of scoping reviews. JBI Evid. Synth..

[21] Munn Z., Peters M.D.J., Stern C., Tufanaru C., McArthur A., Aromataris E. (2018). Systematic review or scoping review? Guidance for authors when choosing between a systematic or scoping review approach. BMC Med. Res. Methodol..

[22] Gow S.P. (2007). Investigation of Antimicrobial Resistance and Antimicrobial Use in Western Canadian Cow-Calf Herds. Ph.D. Thesis.

[23] Agga G.E., Schmidt J.W., Arthur T.A. (2016). Antimicrobial-resistant fecal bacteria from ceftiofur-treated and nonantimicrobial-treated comingled beef cows at a cow–calf operation. Microb. Drug Res..

[24] Beef 2007–08 Antimicrobial Drug Use and Antimicrobial Resistance on U.S. Cow-Calf Operations, 2007–2008. https://www.aphis.usda.gov/animal_health/nahms/beefcowcalf/downloads/beef0708/Beef0708_ir_Antimicrobial_1.pdf.

[25] Schmidt J.W., Vikram A., Arthur T.A., Belk K.E., Morley P.A., Weinroth M.D., Wheeler T.L. (2020). Antimicrobial resistance at two U.S. cull cow processing establishments. J. Food Protect..

[26] Nobrega N., Andres-Lasheras S., Zaheer R., McAllister T., Homerosky E., Anholt R.M., Dorin C. (2021). Prevalence, risk factors, and antimicrobial resistance profile of respiratory pathogens isolated from suckling beef calves to reprocessing at the feedlot: A longitudinal study. Front. Vet. Sci..

[27] Guo Y., McMullen C., Timsit E., Hallewell J., Orsel K., van der Meere F., Yan S., Alexander T.W. (2020). Genetic relatedness and antimicrobial resistance in respiratory bacteria from beef calves sampled from spring processing to 40 days after feedlot entry. Vet. Microbiol..

[28] Beef Cow-Calf Management Practices in the United States, 2017, Report 1. https://www.aphis.usda.gov/animal_health/nahms/beefcowcalf/downloads/beef2017/Beef2017_dr_PartI.pdf.

[29] Markland S., Weppelmann T.A., Ma Z., Lee S., Mir R.A., Teng L., Ginn A., Lee C., Ukhanova M., Galindo S. (2019). High prevalence of cefotaxime resistant bacteria in grazing beef cattle: A cross sectional study. Front. Microbiol..

[30] Waldner C.L., Gow S., Parker S., Campbell J.R. (2019). Antimicrobial resistance in fecal *Escherichia coli* and *Campylobacter* spp. from beef cows in western Canada and associations with herd attributes and antimicrobial use. Can. J. Vet. Res..

[31] Waldner C.L., Parker S., Gow S., Wilson D.J., Campbell J.R. (2019). Antimicrobial usage in western Canadian cow-calf herds. Can. Vet. J..

[32] Waldner C.L., Parker S., Gow S., Wilson D.J., Campbell J.R. (2019). Attitudes towards antimicrobial use and factors associated with antimicrobial use in western Canadian cow-calf herds. Can. Vet. J..

[33] Waldner C., Jelinski M.D., McIntyre-Zimmer K. (2013). Survey of western Canadian beef producers regarding calf-hood diseases, management practices, and veterinary service usage. Can. Vet. J..

[34] Gow S.P., Waldner C.L., Rajic A., McFall M.E., Reid-Smith R. (2008). Prevalence of antimicrobial resistance in fecal generic *Escherichia coli* isolated in western Canadian beef herds. Part II—Cows and cow-calf pairs. Can. J. Vet. Res..

[35] Bae W., Kaya K.N., Hancock D.D., Call D.R., Park Y.H., Besser T.E. (2005). Prevalence and antimicrobial resistance of thermophilic *Campylobacter* spp. from cattle farms in Washington State. Appl. Environ. Microbiol..

[36] Gow S.P., Waldner C.L., Rajic A., McFall M.E., Reid-Smith R. (2008). Prevalence of antimicrobial resistance in fecal generic *Escherichia coli* isolated in western Canadian cow-calf herds. Part I—Beef calves. Can. J. Vet. Res..

[37] Gow S.P., Waldner C.L. (2009). Antimicrobial drug use and reason for treatment in 203 western Canadian cow–calf herds during calving season. Prev. Vet. Med..

[38] Gow S.P., Waldner C.L. (2009). Antimicrobial resistance and virulence factors stx1, stx2, and eae in generic *Escherichia coli* isolates from calves in western Canadian cow-calf herds. Microb. Drug Res..

[39] Berge A.C., Hancock D.D., Sischo W.M., Besser T.E. (2010). Geographic, farm, and animal factors associated with multiple antimicrobial resistance in fecal *Escherichia coli* isolates from cattle in the western United States. J. Am. Vet. Med. Assoc..

[40] Doidge C., Dickie J., Lovatt F., Hudson C., Kaler J. (2021). Evaluation of the use of antibiotic waste bins and medicine records to quantify antibiotic use on sheep, beef, and mixed species farms: A mixed methods study. Prev. Vet. Med..

[41] Hope K.J., Apley M.D., Schrag N.F.D., Lubbers B.V., Singer R.S. (2020). Comparison of surveys and use records for quantifying medically important antimicrobial use in 18 U.S. beef feedyards. Zoonoses Public Health.

[42] Baptiste K.E. (2003). Associations of Penicillin-Resistant *Staphylococcus aureus* and β-Lactam Drug Usage in Danish Dairy Herds. Ph.D. Thesis.

[43] Collineau L., Belloc C., Stärk K.D.C., Hémonic A., Postma M., Dewulf J., Chauvin C. (2016). Guidance on the selection of appropriate indicators for quantification of antimicrobial usage in humans and animals. Zoonoses Public Health.

[44] Gozdzielewska L., King C., Flowers P., Mellor D., Dunlop P., Price L. (2020). Scoping review of approaches for improving antimicrobial stewardship in livestock farmers and veterinarians. Prev. Vet. Med..

[45] Waldner C., Wilhelm B., Windeyer C., Parker S., Campbell J. (2022). Improving beef calf health: Frequency of disease syndromes, uptake of management practices following calving, and potential for antimicrobial use reduction in western Canadian herds. Trans. An. Sci..

[46] Canadian Integrated Program for Antimicrobial Resistance Surveillance (CIPARS) 2018: Design and Methods. https://www.canada.ca/en/public-health/services/surveillance/canadian-integrated-program-antimicrobial-resistance-surveillance-cipars/cipars-reports/2018-annual-report-design-methods.html.

[47] Understanding Susceptibility Test Data as a Component of Antimicrobial Stewardship in Veterinary Settings. https://clsi.org/standards/products/veterinary-medicine/documents/vet09/.

[48] Morris C., Wickramasingha D., Abdelfattah E.M., Pereira R.V., Okello E., Maier G. (2023). Prevalence of antimicrobial resistance in fecal *Escherichia coli* and *Enterococcus* spp. isolates from beef cow-calf operations in northern California and associations with farm practices. Front. Microbiol..

[49] Waldner C.L. (2008). Western Canada study of animal health effects associated with exposure to emissions from oil and natural gas field facilities. Study design and data collection. 1. Herd performance records and management. Arch. Environ. Occ. Health..

[50] Baker S.J., Payne D.J., Rappuoli R., De Gregorio E. (2018). Technologies to address antimicrobial resistance. Proc. Natl. Acad. Sci. USA.

[51] Amat S., Timsit E., Workentine M., Schwinghamer T., van der Meer F., Guo Y., Alexander T.W. (2023). A single intranasal dose of bacterial therapeutics to calves confers longitudinal modulation of the nasopharyngeal microbiota: A pilot study. mSystems.

[52] Redweik G.A.J., Horak M.K., Hoven R., Ott L., Mellata M. (2021). Evaluation of live bacterial prophylactics to decrease IncF plasmid transfer and association with intestinal small RNAs. Front. Microbiol..

[53] Ross J., Schatz C., Beaugrand K., Zuidhof S., Ralston B., Allan N., Olson M. (2021). Evaluation of activated charcoal as an alternative to antimicrobials for the treatment of neonatal calf diarrhea. Vet. Med..

[54] Carter H.M.S., Steele M.A., Costa J.H.C., Renaud D.L. (2022). Evaluating the effectiveness of colostrum as a therapy for diarrhea in preweaned calves. J. Dairy Sci..

[55] Fan P., Nelson C.D., Driver J.D., Elzo M.A., Jeong K.C. (2019). Animal breed composition is associated with the hindgut microbiota structure and beta-lactam resistance in the multibreed Angus-Brahman herd. Front. Microbiol..

[56] Lee S., Teng L., DiLorenzo N., Weppelmann T.A., Jeong K.C. (2020). Prevalence and molecular characteristics of extended-epectrum and AmpC b-Lactamase producing *Escherichia coli* in grazing beef cattle. Front. Microbiol..

[57] Stroup D.F., Berlin J.A., Morton S.C., Olkin I., Williamson G.D., Rennie D., Moher D., Becker B.J., Sipe T.A., Thacker S.B. (2000). Meta-analysis of observational studies in epidemiology: A proposal for reporting. Meta-analysis Of Observational Studies in Epidemiology (MOOSE) group. J. Am. Med. Assoc..

[58] Mueller M., D’Addario M., Egger M., Cevallos M., Dekkers O., Mugglin C., Scott P. (2018). Methods to systematically review and meta-analyse observational studies: A systematic scoping review of recommendations. BMC Med. Res. Methodol..

[59] Tricco A.C., Lillie E., Zarin W., O’Brien K.K., Colquhoun H., Levac D., Moher D., Peters M.D.J., Horsley T., Weeks L. (2018). PRISMA Extension for Scoping Reviews (PRISMA-ScR): Checklist and Explanation. Ann. Int. Med..

[60] Munn Z., Sandeep Moola S., Lisy K., Riitano D., Tufanaru C. (2015). Methodological guidance for systematic reviews of observational epidemiological studies reporting prevalence and cumulative incidence data. Int. J. Evid. Based Healthc..

