# Supplementary material for: A Scoping Review of Antimicrobial Usage and Antimicrobial Resistance in Beef Cow–Calf Herds in the United States and Canada
Source: Antibiotics (Basel). 2023 Jul 12;12(7):1177. doi: 10.3390/antibiotics12071177 (PMC10376086; doi:10.3390/antibiotics12071177)
Supplement: Supplementary file 1 [file antibiotics-12-01177-s001.zip › antibiotics-2457377-supplementary -final.pdf]

antibiotics

MDPI

File S1: Protocol: Scoping review of antimicrobial use and antimicrobial resistance in the North American cow–calf sector

Background

Antimicrobial resistance (AMR) is an important issue for the North American beef cow–calf industry due to its potential impact on treatment failures and resulting animal welfare issues, and its influence on consumer perception of the industry. While antimicrobial usage (AMU) is widely recognized as a driver of AMR, describing AMU, AMR, and risk factors for AMU and AMR is necessary to inform antimicrobial stewardship initiatives.

Therefore, the objectives of this review are:

- Map AMU/AMR research conducted in the North American beef cow–calf sector.
- Characterize AMU/AMR and predictors in the beef cow–calf sector.
- Identify research gaps pertinent to the development of AMR risk mitigation strategies.

Methods

This scoping review will be conducted following a protocol informed by Joanna Briggs Institute (JBI) Evidence Synthesis [1] and the Preferred Reporting Items for Systematic Reviews with Extension for Scoping Reviews [2].

Eligibility Criteria

Given the aim of the review, the condition, context, and population (CoCoPop acronym) described by [3] were used to characterize eligibility criteria. The criteria are outlined as follows:

Condition: Antimicrobial use or antimicrobial resistance, or risk factors potentially associated with either of these, reported in any metric. Metrics for describing AMU may vary across studies and all are deemed relevant, including count-, weight-, or dose-based.

Context: Environmental factors can have an impact on the frequency of occurrence of the conditions of interest [3], and these factors (e. g. geographic location and season), were included when reported by authors.

Population: Studies with healthy/asymptomatic animals produced in beef cow–calf herds in Canada and the U. S. were eligible. Studies of all classes of livestock within the cow–calf sector (pre-weaning calves, breeding heifers and cows, breeding bulls) were eligible. For studies reporting data from cow–calf operations as well as other enterprises such as stockers, backgrounding or feedlots, or studies sampling operations which combined one or more of these enterprises with cow–calf production, sampling from the populations of interest was deemed relevant, and sampling originating from populations outside of scope (weaned calves or other feeder animals) was not included in data extraction.

Inclusion: Primary research investigating beef cow–calf herds in Canada and the U.S., of any size, was eligible with no restrictions on year or language of publication. If the research included AMR outcomes, only data originating from sampling clinically healthy as opposed to ill animals

were deemed relevant. Journal articles, government or industry publications, and theses were all deemed relevant.

Exclusion: Studies conducted in countries other than Canada and the U. S. were excluded to restrict data to regionally relevant herd management practices, licensed antimicrobials, and regulations limiting access. Additionally, AMR studies were excluded if they only sampled clinically ill or symptomatic animals, or they did not report outcomes identifiable as originating from clinically healthy animals or collected as part of routine surveillance from privately owned herds. Calf rearing operations, in which dairy calves not destined for the breeding herd are fed to slaughter, were outside scope, even if reporting on calves of beef breeds. Studies investigating cattle in feedlots or only sampling cattle raised within organic or antibiotic-free production systems were also deemed outside of scope.

Search

A search was conducted in four electronic bibliographic databases: Web of Science, Scopus, AGRICOLA, and NCBI Pubmed, using a three-term search. One term describes the populations of interest (e.g., cows, calves, or beef cattle). The second term describes the outcome of interest (e.g., AMR or AMU). The third term describes location (e.g., Canada or U.S.). A search in Google Scholar will also be conducted using the same strategy as employed in the electronic bibliographic databases. Finally, a hand-search of the references section of relevant research studies and reviews captured will be executed to identify papers not captured by the electronic search.

Review Process, Data Cleaning, and Analysis

First-level relevance screening will be performed independently by two reviewers on citations captured by the search. The first level relevance screening form asks four questions: "Is the literature in the form of a journal article, thesis, or government report?"; "Does the research take place in Canada or the United States?"; "Does the study include data from cow-calf herds?"; and "Does the article contain information on AMU or AMR?". Those citations deemed relevant using this form will be promoted to second-level relevance screening, and the full paper obtained.

The objective of second-level relevance screening is a full-text review by two independent reviewers to confirm eligibility. Relevant publications containing information on AMU or AMR in the cow–calf sector of the beef industry, specifically the Canadian or American cow–calf industries, will be promoted.

Third-level data extraction will be performed by a single reviewer using a pre-tested data extraction form capturing major characteristics of each relevant study, such as year of sampling, populations studied, broad outcome(s) reported (i.e. AMU or AMR) and metrics used, as well as level of aggregation of data (isolate/individual/herd).

For purposes of this review, a study is defined as an investigation of AMU or AMR in a defined population, identifiable by beef cow–calf sub-populations (breeding cows, replacement heifers, pre-weaned calves, post-weaned calves, bulls, or ALL animals in the cow–calf herd). A publication is defined as a publicly available description of one or more studies (so synonymous with

“paper”). The same study population could be sampled for different studies (e.g., the same group of herds could be the study population for one study describing AMU, and another describing AMR). Multiple studies could originate from the same overall study population, within one publication (e.g., AMU of a study group of herds could describe breeding cows and calves). For purposes of mapping the literature, all relevant publications will be included in data extraction. If multiple publications sample the same study population for different studies, typically reporting different outcomes, these will be indicated with a superscript in tables.

Descriptive statistics will be computed in a commercial spreadsheet program (Excel™, Microsoft 365, Microsoft Corp).

References

[1]. Peters, M., Marnie, C., Tricco, A.C., Pollock, D., Munn, Z., Alexander, A., McInerney, P., Godfrey, C.M., Khalil, H. Updated methodological guidance for the conduct of scoping reviews. *JBI evidence synthesis* [online]. 2020, 18, 2119-2126. DOI: 10.11124/JBIES-20-00167

[2]. Tricco, A.C., Lillie, E., Zarin, W., O'Brien, K.K., Colquhoun, H., Levac, D., Moher, D., Peters, M.D.J., Horsley, T., Weeks, L., Hempel, S., Akl, E.A., Chang, C., McGowan, J., Stewart, L., Hartling, L., Aldcroft, A., Wilson, M.G., Garritty, C., Lewin, S., Godfrey, C.M., Macdonald, M.T., Langlois, E.V., Soares-Weiser,K., Moriarty, J., Clifford, T., Tunçalp, Ö., Straus, S.E. PRISMA Extension for Scoping Reviews (PRISMA-ScR) : Checklist and Explanation. *Ann. Int. Med.* 2018,169, 467-473. https://doi.org/10.7326/M18-0850

[3]. Munn, Z., Sandeep Moola, S., Lisy, K., Riitano, D., Tufanaru, C., 2015. Methodological guidance for systematic reviews of observational epidemiological studies reporting prevalence and cumulative incidence data. *Int. J. Evid. Based Healthc.* 2015, 13, 147-153. DOI: 10.1097/XEB.0000000000000054

Search strategy

i. Web of Science Search

Line	Search String	Results
1	TS=("cow-calf" OR "pairs" OR "cows" OR "calves" OR "beef cattle")	583,119
2	TS=("antimicrobial " OR AMU OR "antimicrobial resistance" OR AMR)	343,737
3	ALL=("North America" OR Canada OR "United States")	1,138,628
4	1 AND 2 AND 3	329

ALL=(("cow-calf" OR "pairs" OR "cows" OR "calves" OR "beef cattle") AND (antimicrobial NEAR/1 (use OR resistance)) AND ("North America" OR Canada OR "United States")).

ii. Scopus Search

Line	Search String	Results
1	TITLE-ABS-KEY ("cow-calf" OR "pairs" OR "cows" OR "calves" OR "beef cattle")	1,288,086
2	TITLE-ABS-KEY (antimicrobial W/1 (use OR resistance))	48,214
3	TITLE-ABS-KEY ("North America" OR Canada OR "United States")	2,643, 546
4	#1 AND #2 AND #3	126

iii. Agricola Search

Line	Search String	Results
1	("cow-calf" OR "pairs" OR "cows" OR "calves" OR "beef cattle")	154,422
2	antimicrobial NEAR/1 (use OR resistance)	8,292
3	"North America" OR "Canada" OR "United States"	687,818
4	1 AND 2 AND 3	129

iv. NCBI Pubmed Search

Line	Search String	Results
1	("cow-calf" OR "pairs" OR "cows" OR "calves" OR "beef cattle")	28,246
2	antimicrobial (use OR resistance)	4303
3	"North America" OR "Canada" OR "United States"	538,165

4	1 AND 2 AND 3	262

First level relevance screening tool (performed on citation and abstract)

Question	Response options	Comments
• Is the literature in the form of a journal article, thesis, or government report?	a. Yes<br>b. No<br>c. Can't tell	
• Does the research take place in Canada or the United States?	a. Yes<br>b. No<br>c. Can't tell	
• Does the study include data from cow–calf herds?	• Yes<br>• No<br>• Can't tell	Exclusions listed below
• Does the article contain information on AMU or AMR?	a. Yes<br>b. No<br>c. Can't tell	Exclusions listed below

Review process:

IF (1a OR 1c) AND (2a OR 2c) AND (3a OR 3c) AND (4a OR 4c)

THEN procure paper and promote to level 2 screening.

Exclusions: Studies investigating only cattle in feedlots or conducted in countries other than Canada and the U.S., or, if studying AMR, sampling only clinically ill animals, or not reporting outcomes identifiable as originating from clinically healthy animals. Calf rearing

operations, in which dairy calves not destined for the breeding herd are fed to slaughter, are outside of scope, even if reporting on calves of beef breeds. Studies only sampling cattle raised within organic or antibiotic-free production systems, are outside of scope.

Second level relevance screening tool (performed on full paper)

**Question**	**Response options**	**Comments**
• Is the literature in the form of a journal article, thesis, or government report?	a. Yes<br>b. No<br>c. Cannot tell	
• Does the research take place in Canada or the United States?	a. Yes<br>b. No<br>c. Cannot tell	
• Does the study include data from cow–calf herds?	a. Yes<br>b. No<br>c. Cannot tell	Exclusions listed below
• Does the article contain information on AMU or AMR?	a. Yes<br>b. No<br>c. Cannot tell	Exclusions listed below

Review process:

IF (1a) AND (2a) AND (3a) AND (4a)

THEN promote to level 3 data extraction.

Exclusions: Studies investigating only cattle in feedlots or conducted in countries other than Canada and the U.S., or, if studying AMR, sampling only clinically ill animals, or not reporting outcomes identifiable as originating from clinically healthy animals. Calf rearing operations, in which dairy calves not destined for the breeding herd are fed to slaughter, are outside scope, even if reporting on calves of beef breeds. Studies only sampling cattle raised within organic or antibiotic-free production systems, are outside of scope.

Third-level data extraction form

Please note: Data extraction was completed using a horizontal layout; however, the categories were transposed to facilitate easier display in the supplementary material.

**Instructions:**							
Black text column 3 = questions							
Blue text column 4 = answers: select one or more options (as per question), or answer in free text							
Each row to be completed for one discrete group, potentially defined by: population_time_System usage_agent usage_method_drug class-drug							
	**MAJOR DATA CATEGORIES**	**SPECIFIC DATA CATEGORIES**	**ANSWER OPTIONS**	**EXAMPLE DATA EXTRACTION**			
**CITATION**		**Citation**	Free text	Citation 1		Citation 2	
		**Year Published**	Free text	Citation 1 row 1	Etc.	Citation 2 row 1	Etc.
**CITATION ROW DESCRIPTORS**		**State year of sampling/population sampled/other descriptors defining population THIS ROW**	Free text				
		**AMU investigated?**	Y-N				
		**AMR investigated?**	Y-N				
**METHODS**	**Overview participants**	**Location**	Canada_US				
		**Loc_detail**	Prov_state_region				
		**Year(s) of data collection for this study**	Free text				
		**Years of data collection for THIS ROW**	Free text				

		**Operation types investigated this study? Please indicate all that apply.**	Cow–calf_feedlot_combination			
		**Inclusion criteria reported**	Free text			
	**Population**	**Populations studied in this paper? Please indicate all that apply**	Cows_bulls_pre-weaning_post-weaning_replacements_ALL			
		**Population studied THIS ROW**	Cows_bulls_pre-weaning_post-weaning_replacements_ALL			
		**Pairs (cow–calf) studied in this paper?**	Y-N			
**Sampling methods**		**Nature of data captured? Please indicate all that apply.**	Physical/Reported			
		**Instrument type used to capture data? Please indicate all that apply.**	Questionnaire_interview_diary_garbagecan_N/A			
		**Sample Matrix collected? Please indicate all that apply.**	Feces_NP_N/A_Free text			
		**Sample matrix collected THIS ROW**	Feces_NP_N/A_Free text			
		**Sampling scheme as described by authors?**	Free text_paste			
		**Sample Collection plan as described by authors?**	Free text			
		**Season sampled? Please indicate all that apply.**	Spring-Fall-NA-NR			
		**Season sampled THIS ROW**	Spring-Fall-NA-NR			
		**Production year: is a full production year sampled?**	Y-N			
		**Sampling unit: What sampling units are reported in THIS ROW**	Herd-Individual			
		**Number Samples collected per herd**	Free text			
		**Number herds sampled in this investigation**	Free text			
		**Number individuals sampled in this investigation**	Free text - NR			
		**Herds: number sampled THIS ROW**	Free text			
		**Herd size_lowest end of range of herds sampled**	Free text			
		**Herd size_highest end of range sampled**	Free text			
		**Herd size_median of herds sampled**	Free text			
		**Herd size mean of herds sampled**	Free text			
**AMU**		**AMU captured in THIS ROW?**	Y-N			
		**Denom_ total this row: What is the total number of sampling units sampled for THIS ROW?**	Free text			
	**USAGE**	**Ind AMU records available to investigators?**	Y-N			

		Objective of AMU as described by authors THIS ROW?	Respiratory disease-digestive disease- lameness-other-NR-Free text				
		Strategy for objective identified (for value in "Objective of AMU described by authors") THIS ROW	metaphylaxis_prophylaxis_masstx_individualtx-NR-Free text				
		MIA (medically important antimicrobials) discussed?	Y/N				
		Level of data aggregation THIS ROW	herd_group e.g. cows-other Free text				
		Population THIS ROW	cows_calves-replacements- bulls- ALL-NR				
		Number receiving AM THIS ROW	Free text				
		Range_lo (for value in "Level of data aggregation") if summary measure	Free text				
		Range_hi if summary measure	Free text				
		Median if summary measure	Free text				
		Mean if summary measure	Free text				
		95%CI_low if summary measure	Free text				
		95%CI-high if summary measure	Free text				
DRUGS USED		Administration route of antimicrobials THIS ROW	Feed_Water_Oral_Inj_Topical_Other				
		Metric for AMU THIS ROW	Count_Dosage_Weight_free text				
		Time interval captured for usage THIS ROW	Time interval captured				
		Dose used in animals this row reported?	Y/N				
	Drug: for each pertinent drug, in free text report AMU (count proportion, etc., as previously indicated),	Any	Free text				
		(CI)	Free text				
		None	Free text				
		(CI)	Free text				
		Ampicillin	Free text				
		(CI)	Free text				
		Amphenicols	Free text				
		(CI)	Free text				
		Amp-sulbactam	Free text				

		**(CI)**	Free text				
		**Aminoglycosides**	Free text				
		**(CI)**	Free text				
		**Aminocyclitol**	Free text				
		**(CI)**	Free text				
		**Benzyl penicillin procaine**	Free text				
		**(CI)**	Free text				
		**Benzyl penicillin procaine/benzathine**	Free text				
		**(CI)**	Free text				
		**Beta lactams**	Free text				
		**(CI)**	Free text				
		**Ceftiofur**	Free text				
		**(CI)**	Free text				
		**Ceftiofur crystalline free acid**	Free text				
		**(CI)**	Free text				
		**Ceftiofur hydrochloride**	Free text				
		**(CI)**	Free text				
		**Ceftiofur sodium**	Free text				
		**(CI)**	Free text				
		**Cephalosporins**	Free text				
		**(CI)**	Free text				
		**Cephapirin sodium**	Free text				
		**(CI)**	Free text				
		**Chlortetracycline**	Free text				
		**(CI)**	Free text				
		**Chlor-sulfamethazine**	Free text				
		**(CI)**	Free text				
		**Combinations**	Free text				
		**Danofloxacin**	Free text				

		**(CI)**	Free text				
		**Decoquinate-ionophores**	Free text				
		**(CI)**	Free text				
		**Dihydrostreptomycin**	Free text				
		**(CI)**	Free text				
		**Enrofloxacin**	Free text				
		**(CI)**	Free text				
		**Erythromycin**	Free text				
		**(CI)**	Free text				
		**Florfenicol**	Free text				
		**(CI)**	Free text				
		**Flor+flunixin**	Free text				
		**(CI)**	Free text				
		**Fluoroquinolones**	Free text				
		**(CI)**	Free text				
		**Folate inhibitor**	Free text				
		**(CI)**	Free text				
		**Gamithromycin**	Free text				
		**(CI)**	Free text				
		**Gentamicin**	Free text				
		**(CI)**	Free text				
		**Lasalocid**	Free text				
		**(CI)**	Free text				
		**Lincomycin-spectinomycin**	Free text				
		**(CI)**	Free text				
		**Macrolides**	Free text				
		**(CI)**	Free text				
		**Monensin**	Free text				
		**(CI)**	Free text				

		**Non-cephalosporin beta lactams**	Free text				
		**(CI)**	Free text				
		**Neomycin**	Free text				
		**(CI)**	Free text				
		**Oxytetracycline**	Free text				
		**(CI)**	Free text				
		**Penicillin**	Free text				
		**(CI)**	Free text				
		**Penicillin G procaine**	Free text				
		**(CI)**	Free text				
		**Polymixin B**	Free text				
		**(CI)**	Free text				
		**Peptides**	Free text				
		**(CI)**	Free text				
		**Phenicols**	Free text				
		**(CI)**	Free text				
		**Quinolones**	Free text				
		**(CI)**	Free text				
		**Spectinomycin**	Free text				
		**(CI)**	Free text				
		**Sulfonamides**	Free text				
		**(CI)**	Free text				
		**Sulfamethazine**	Free text				
		**(CI)**	Free text				
		**Tetracycline**	Free text				
		**(CI)**	Free text				
		**TetHCl**	Free text				
		**(CI)**	Free text				
		**Tiamulin**	Free text				

		**(CI)**	Free text				
		**Tildipirosin**	Free text				
		**(CI)**	Free text				
		**Tilmicosin**	Free text				
		**(CI)**	Free text				
		**TMS**	Free text				
		**Tulathromycin**	Free text				
		**(CI)**	Free text				
		**Tylosin**	Free text				
		**(CI)**	Free text				
	RESISTANCE	**AMR data reported?**	Y-N				
		**Duration of previous treatment(s) on animals THIS ROW reported?**	Free text				
		**Bacterial species investigated THIS ROW**	Free text				
		**Repeated measures reported on subjects**	Y-N				
		**Repeated measures detail**	Free text				
		**Paired measures reported? i.e. outcomes identifiable to cow–calf pair?**	Y-N				
		**Paired measures detail**	Free text				
		**Outcome Measure THIS ROW**	Count_Proportion				
		**Number samples THIS ROW**	Free text				
		**Aggregation level of data THIS ROW**	Isolate_Ind_Group_Herd				
		**AST method**	Dis diffusion_Sensititre_free text				
		**Breakpoints reported?**	CLSI-NCLSS_NARMS_free text				
		**MICs reported?**	Y-N				
		**Herd_number THIS ROW**	Free text				
		**Ind_num THIS ROW**	Free text				
		**Isolate_num THIS ROW**	Free text				
	**DRUGS: for each pertinent drug, in free text report AMR (count propor-tion, etc., as previously**	**None**	Free text				

	**indicated)**						
		**CI**	Free text				
		**Any**	Free text				
		**(CI)**	Free text				
		**Aminoglycosides**	Free text				
		**(CI)**	Free text				
		**Penicillins**	Free text				
		**(CI)**	Free text				
		**Beta lactams**	Free text				
		**(CI)**	Free text				
		**3rd gen cephalosporins**	Free text				
		**(CI)**	Free text				
		**Folic acid inhibitors**	Free text				
		**(CI)**	Free text				
		**Macrolides**	Free text				
		**(CI)**	Free text				
		**Phenicols**	Free text				
		**(CI)**	Free text				
		**Quinolones**	Free text				
		**(CI)**	Free text				
		**Sulfas**	Free text				
		**(CI)**	Free text				
		**Amikacin**	Free text				
		**(CI)**	Free text				
		**Amoxicillin-clavulanic acid**	Free text				
		**(CI)**	Free text				
		**Ampicillin**	Free text				
		**(CI)**	Free text				
		**Azithromycin**	Free text				

		**(CI)**	Free text				
		**Cefoxitin**	Free text				
		**(CI)**	Free text				
		**Ceftiofur**	Free text				
		**(CI)**	Free text				
		**Ceftriaxone**	Free text				
		**(CI)**	Free text				
		**Cefoxatime**	Free text				
		**(CI)**	Free text				
		**Cephalothin**	Free text				
		**(CI)**	Free text				
		**Chloramphenicol**	Free text				
		**(CI)**	Free text				
		**Cipro**	Free text				
		**(CI)**	Free text				
		**Cefoxitin**	Free text				
		**(CI)**	Free text				
		**Clindamycin**	Free text				
		**(CI)**	Free text				
		**Danofloxacin**	Free text				
		**(CI)**	Free text				
		**Enrofloxacin**	Free text				
		**(CI)**	Free text				
		**Erythromycin**	Free text				
		**(CI)**	Free text				
		**Florfenicol**	Free text				
		**(CI)**	Free text				
		**Gamithromycin**	Free text				
		**(CI)**	Free text				

		**Gentamicin**	Free text				
		**(CI)**	Free text				
		**Kanamycin**	Free text				
		**(CI)**	Free text				
		**Levofloxacin**	Free text				
		**(CI)**	Free text				
		**Linezolid**	Free text				
		**(CI)**	Free text				
		**Metronidazole**	Free text				
		**(CI)**	Free text				
		**Nalidixic acid**	Free text				
		**(CI)**	Free text				
		**Neomycin**	Free text				
		**(CI)**	Free text				
		**Oxytet**	Free text				
		**(CI)**	Free text				
		**Penicillin**	Free text				
		**(CI)**	Free text				
		**Rifampicin**	Free text				
		**(CI)**	Free text				
		**Streptomycin**	Free text				
		**(CI)**	Free text				
		**Sulfamethoxazole**	Free text				
		**(CI)**	Free text				
		**Sulfisoxazole**	Free text				
		**(CI)**	Free text				
		**Telithromycin**	Free text				
		**(CI)**	Free text				
		**Tetracycline**	Free text				

		**(CI)**	Free text				
		**Tildipirosin**	Free text				
		**(CI)**	Free text				
		**Tilmicosin**	Free text				
		**(CI)**	Free text				
		**TMS**	Free text				
		**(CI)**	Free text				
		**Tulathromycin**	Free text				
		**(CI)**	Free text				
		**Tylosin**	Free text				
		**(CI)**	Free text				
		**Vancomycin**	Free text				
		**(CI)**	Free text				
		**Min 1**	Free text				
		**(CI)**	Free text				
		**Min 2**	Free text				
		**(CI)**	Free text				
		**Min 3**	Free text				
		**(CI)**	Free text				
		**Min 4**	Free text				
		**(CI)**	Free text				
		**Min 5**	Free text				
		**(CI)**	Free text				
		**Comments**	Free text				
	**RISK FACTORS**	**re AMU**	Y-N				
		**re AMR**	Y-N				
		**Risk Factor investigated**	Free text				
		**Outcome measured**	Free text				
		**Risk factor level aggregated**	Herd-Individual-Other Free text				

		**Outcome level aggregated**	Herd-Individual-Other Free text				
	**MEASURE OF ASSOCIATION**	**Odds Ratio**					
		**CI low**					
		**CI high**					
		***P* value**					
		**COMMENTS**					

Abbreviations: AMU = antimicrobial use; AMR = antimicrobial resistance; CI = Confidence Intervals; N/A = not applicable; NR = Not reported; Y = Yes; N = No

Preferred Reporting Items for Systematic Reviews and Meta-Analyses Extension for Scoping Reviews (PRISMA-ScR) Checklist

SECTION	ITEM	PRISMA-ScR CHECKLIST ITEM	REPORTED ON PAGE #
Title	1	Identify the report as a scoping review.	1
Structured summary	2	Provide a structured summary that includes (as applicable): background, objectives, eligibility criteria, sources of evidence, charting methods, results, and conclusions that relate to the review questions and objectives.	1
Rationale	3	Describe the rationale for the review in the context of what is already known. Explain why the review questions/objectives lend themselves to a scoping review approach.	1-2
Objectives	4	Provide an explicit statement of the questions and objectives being addressed with reference to their key elements (e.g., population or participants, concepts, and context) or other relevant key elements used to conceptualize the review questions and/or objectives.	3
Protocol and registration	5	Indicate whether a review protocol exists; state if and where it can be accessed (e.g., a Web address); and if available, provide registration information, including the registration number.	File S1
Eligibility criteria	6	Specify characteristics of the sources of evidence used as eligibility criteria (e.g., years considered, language, and publication status), and provide a rationale.	20-21
Information sources[1]	7	Describe all information sources in the search (e.g., databases with dates of coverage and contact with authors to identify additional sources), as well as the date the most recent search was executed.	21
Search	8	Present the full electronic search strategy for at least 1 database, including any limits used, such that it could be repeated.	21, File S1 p. 4

Selection of sources of evidence[2]	9	State the process for selecting sources of evidence (i.e., screening and eligibility) included in the scoping review.	21
Data charting process	10	Describe the methods of charting data from the included sources of evidence (e.g., calibrated forms or forms that have been tested by the team before their use, and whether data charting was performed independently or in duplicate) and any processes for obtaining and confirming data from investigators.	21-22
Data items	11	List and define all variables for which data were sought and any assumptions and simplifications made.	File S1 pp. 7-18
Critical appraisal of individual sources of evidence[3]	12	If done, provide a rationale for conducting a critical appraisal of included sources of evidence; describe the methods used and how this information was used in any data synthesis (if appropriate).	N/A
Synthesis of results	13	Describe the methods of handling and summarizing the data that were charted.	22
Selection of sources of evidence	14	Give numbers of sources of evidence screened, assessed for eligibility, and included in the review, with reasons for exclusions at each stage, ideally using a flow diagram.	20-22, Fig. 1
Characteristics of sources of evidence	15	For each source of evidence, present characteristics for which data were charted and provide the citations.	Tables 1-3
Critical appraisal within sources of evidence	16	If completed, present data on critical appraisal of included sources of evidence (see item 12).	N/A
Results of individual sources of evidence	17	For each included source of evidence, present the relevant data that were charted that relate to the review questions and objectives.	Tables 1-3, Tables S1-4<br>3-20
Synthesis of results	18	Summarize and/or present the charting results as they relate to the review questions and objectives.	3-20
Summary of evidence	19	Summarize the main results (including an overview of concepts, themes, and types of evidence available), link to the review questions and objectives, and consider the relevance to key groups.	16-20

Limitations	20	Discuss the limitations of the scoping review process.	20
Conclusions	21	Provide a general interpretation of the results with respect to the review questions and objectives, as well as potential implications and/or next steps.	22
Funding	22	Describe sources of funding for the included sources of evidence, as well as sources of funding for the scoping review. Describe the role of the funders of the scoping review.	22

[1] Where *sources of evidence* (see second footnote) are compiled from, such as bibliographic databases, social media platforms, and Web sites.

[2] A more inclusive/heterogeneous term used to account for the different types of evidence or data sources (e.g., quantitative and/or qualitative research, expert opinion, and policy documents) that may be eligible in a scoping review as opposed to only studies. This is not to be confused with *information sources* (see first footnote).
[3] The frameworks by Arksey and O'Malley (6) and Levac and colleagues (7) and the JBI guidance (4, 5) refer to the process of data extraction in a scoping review as data charting.
[3] The process of systematically examining research evidence to assess its validity, results, and relevance before using it to inform a decision. This term is used for items 12 and 19 instead of "risk of bias" (which is more applicable to systematic reviews of interventions) to include and acknowledge the various sources of evidence that may be used in a scoping review (e.g., quantitative and/or qualitative research, expert opinion, and policy document).

Abbreviations:
JBI = Joanna Briggs Institute; N/A = not applicable; PRISMA-ScR = Preferred Reporting Items for Systematic Reviews and Meta-Analyses Extension for Scoping Reviews.

Reference:

Tricco AC, Lillie E, Zarin W, O'Brien KK, Colquhoun H, Levac D. et al. 2018. PRISMA Extension for Scoping Reviews (PRISMA-ScR) : Checklist and Explanation. Ann Int Med 2018;169:467-473. https://doi.org/10.7326/M18-0850

Conflict resolution log:

- Regarding inclusion or exclusion of Bae et al. (2005) based upon unique outcome measure (resistance index). Decision to include this study was confirmed by the senior author (CW).

References included in scoping review:

Agga, G.E., Schmidt, J.W., Arthur, T.A. Antimicrobial-resistant fecal bacteria from ceftiofur-treated and nonantimicrobial-treated comingled beef cows at a cow–calf operation. *Microb. Drug Res.* 2016, 2, 598 -608. https://doi-org.cyber.usask.ca/10.1089/mdr.2015.0259S

Bae, W., Kaya, K.N., Hancock, D.D., Call, D.R., Park, Y.H., Besser, T.E. Prevalence and antimicrobial resistance of thermophilic *Campylobacter* spp. from cattle farms in Washington State. *Appl. Environ. Microbiol.* 2005, 71, 169–174. DOI: 10.1128/AEM.71.1.169-174.2005

Beef Cow-calf Management Practices in the United States, 2017, report 1. Available online: https://www.aphis.usda.gov/animal_health/nahms/beefcowcalf/downloads/beef2017/Beef2017_dr_PartI.pdf (accessed on 27 May, 2022).

Beef cow-calf health and management practices in the United States, 2017, report 2. Available online: https://www.aphis.usda.gov/animal_health/nahms/beefcowcalf/downloads/beef2017/beef-2017-part2.pdf (accessed on 27 May, 2022).

Beef 2007–08 Antimicrobial Drug Use and Antimicrobial Resistance on U.S. Cow-calf Operations, 2007–08. Available online: https://www.aphis.usda.gov/animal_health/nahms/beefcowcalf/downloads/beef0708/Beef0708_ir_Antimicrobial_1.pdf (accessed on 27 May 2023).

Berge, A.C., Hancock, D.D., Sischo, W.M., Besser, T.E.. Geographic, farm, and animal factors associated with multiple antimicrobial resistance in fecal *Escherichia coli* isolates from cattle in the western United States. J. Am. Vet. Med. Assoc. 2010; 236: 1338-1344.

Carson, C.A., Reid-Smith, R., Irwin, R.J., Martin, W.S., McEwen, S.A. Antimicrobial resistance in generic fecal *Escherichia coli* from 29 beef farms in Ontario. *Can. J. Vet. Res.* 2008, 72, 119-128.

Carson, C.A., Reid-Smith, R., Irwin, R.J., Martin, W.S., McEwen, S.A. Antimicrobial use on 24 beef farms in Ontario. *Can. J. Vet. Res.* 2008, 72,109-118.

Ekakoro, J.E., Caldwell, M., Strand, E.B., Strickland, L., Okafor, C.C. A survey of antimicrobial use practices of Tennessee beef producers. *BMC Vet. Res*. 2019, 15, 222-233. DOI: 10.1186/s12917-019-1978-6

Fossen, J.D.; Campbell, J.R.; Gow, S.P.; Erickson, N.;Waldner, C.L. Antimicrobial Use in Canadian Cow–Calf Herds. Vet. Sci. **2023**, 10, 366. https://doi.org/10.3390/vetsci10050366

Gow, S.P., Waldner, C.L., Rajic, A., McFall, M.E., Reid-Smith, R. Prevalence of antimicrobial resistance in fecal generic *Escherichia coli* isolated in western Canadian beef herds. Part II — Cows and cow-calf pairs. *Can. J. Vet. Res.* 2008, 72, 91-100

Gow, S.P., Waldner, C.L., Rajic, A., McFall, M.E., Reid-Smith, R. Prevalence of antimicrobial resistance in fecal generic *Escherichia coli* isolated in western Canadian cow-calf herds. Part I — Beef calves. Can. J. Vet. Res. 2008, 72, 82-90.

Gow, S.P., Waldner, C.L. Antimicrobial drug use and reason for treatment in 203 western Canadian cow–calf herds during calving season. *Prev. Vet. Med.* 2009, 90, 55-65. DOI:10.1016/j.prevetmed.2009.03.010

Gow, S.P., Waldner, C.L. Antimicrobial resistance and virulence factors stx1, stx2, and eae in generic *Escherichia coli* isolates from calves in western Canadian cow-calf herds. *Microb. Drug Res.* 2009, 15, 61-67. https://doi-org.cyber.usask.ca/10.1089/mdr.2009.0860

Green, A.L., Carpenter, L.R., Edmisson, D.E., Lane, C.D., Welborn, M.G., Hopkins, F.M., Bemis, D.A., Dunn, J.R. Producer attitudes and practices related to antimicrobial use in beef cattle in Tennessee. *J. Am. Vet. Med. Assoc.* 2010, 237, 1292-1298. https://doi.org/10.2460/javma.237.11.1292

Guo, Y., McMullen, C., Timsit, E., Hallewell, J., Orsel, K., van der Meere, F., Yan, S., Alexander, T.W. Genetic relatedness and antimicrobial resistance in respiratory bacteria from beef calves sampled from spring processing to 40 days after feedlot entry. *Vet. Microbiol.* 2020, 240. https://doi.org/10.1016/j.vetmic.2019.108478

Markland, S., Weppelmann,T.A., Ma, Z., Lee, S., Mir, R.A., Teng, L., Ginn, A., Lee, C., Ukhanova, M., Galindo, S., Carr, C., DiLorenzo, N., Ahn, S., Mah, J-H, Kim, H-Y, Mai, V., Mobley, R., Morris, J.G., Jeong, K.C.C. High prevalence of cefotaxime resistant bacteria in grazing beef cattle: a cross sectional study. *Front. Microbiol*. 2019, 10, 176 DOI:https://doi.org/10.3389/fmicb.2019.00176.

Nobrega, N., Andres-Lasheras, S., Zaheer, R., McAllister, T., Homerosky, E., Anholt, R.M., Dorin, C. Prevalence, risk factors, and antimicrobial resistance profile of respiratory pathogens isolated from suckling beef calves to reprocessing at the feedlot: a longitudinal study. *Front. Vet Sci.* 2021, 8, 764701. DOI: 10.3389/fvets.2021.764701

Schmidt, J.W., Vikram, A., Arthur, T.A., Belk, K.E., Morley, P.A., Weinroth, M.D., Wheeler, T.L. Antimicrobial resistance at two U.S. cull cow processing establishments. *J. Food Protect.* 2020, 83, 2216–2228. https://doi.org/10.4315/JFP-20-2016–2228.

Waldner, C., Jelinski, M.D., McIntyre-Zimmer, K. Survey of western Canadian beef producers regarding calf-hood diseases, management practices, and veterinary service usage. *Can. Vet. J*. 2013, 54, 559–564.

Waldner, C.L., Gow, S., Parker, S., Campbell, J.R. Antimicrobial resistance in fecal *Escherichia coli* and *Campylobacter* spp. from beef cows in western Canada and associations with herd attributes and antimicrobial use. *Can. J. Vet. Res.* 2019, 83, 80-89.

Waldner, C.L., Parker, S., Gow, S., Wilson, D.J., Campbell, J.R. Antimicrobial usage in western Canadian cow-calf herds. *Can. Vet. J.* 2019, 60, 255–267.

Waldner, C.L., Parker, S., Gow, S., Wilson, D.J., Campbell, J.R. Attitudes towards antimicrobial use and factors associated with antimicrobial use in western Canadian cow-calf herds. *Can. Vet. J.* 2019, 60, 391–398.

Table S1. Descriptive summary of selected herd-level outcomes of 9 included antimicrobial use studies in beef cow–calf herds in the United States and Canada

Citation	Population[1]	Sample year	Number of herds	Percentage of herds reporting use (number reporting/total herds): Macrolides	β Lactams: Cephalosporins	Fluoroquinolones	Florfenicol	β Lactams: Penam Penicillins and Other β Lactams	Tetracyclines	Sulfonamides	Diaminopyrimidines-sulfonamides
[1] [2,5]	Cows	2019-2020	146	2.1%[TILD] (3/138)	8.9%[CEFT] (13/138)	0.7%[DANO] (1/138)	16% (24/138)	1.4%[AMP] (2/138)	71%[OXY] (104/138)	0.7%[SANI] (1/138)	9.6%[TSDOX] (14/138)
[1] [2,5]	Cows	2019-2020	146	9.6%[TILM] (14/138)	2.1%[CEPH] (3/138)			27% [BPrP] (39/138)			
[1] [2,5]	Cows	2019-2020	146	10%[TULA] (15/138)				19% [BPrPB] (28/138)			
[1] [2,5]	Calves	2019-2020	138	2.7%[TILD] (4/138)	13%[CEFT] (19/138)	2.7%[DANO] (4/138)	60% (87/138)	1.4%[AMP] (2/138)	53%[OXY] (77/138)	25%[SMT] (37/138)	32%[TSDOX] (46/138)
[1] [2,5]	Calves	2019-2020	138	13%[TILM] (19/138)		2.1%[ENRO] (3/138)		21% [BPrP] (30/138)		12%[SGUA] (18/138)	
[1] [2,5]	Calves	2019-2020	138	24%[TULA] (35/138)				11% [BPrPB] (16/138)			
[2][3,5]	All	2018	88	5% (4/88)	1% (1/88)	1% (1/88)	11% (10/88)	5. 7% (5/88)	66% (58/88)	1% (1/88)	
[3][3,8,11]	Calves	2017	2159	6.5%	1.6%	0.9%	3.6%	5.1%	17.5%		
[3] [3,8,11]	Heifers	2017	2159	4.9%	0	0	0.9%	0.2%	7.2%		

Citation	Popu-lation[1]	Sample year	Num-ber	Percentage of herds reporting use (number reporting/total herds)							
[3] [3,8,11]	Cows	2017	2159	3.7%	1.6%	0	2.1%	1.8%	18.4%		
[3] [3,7,11]	Calves	2017	2159						3.8%	7.1%	
[3] [3,7,11]	Heifers	2017	2159						0	0.7%	
[3] [3,7,11]	Cows	2017	2159						0.7%	3.0%	
[4][2-3,5]	Cows	2013-<br>2014	98	19%<br>(19/98)	3%[FGEN]<br>(3/98)	N/R[3]	28%[FLOR]<br>(27/98)	37%<br>(36/98)	80%<br>(78/98)		7% [TSDOX]<br>(7/98)
[4][2-3,5]	Cows	2013-<br>2014	98		7%[TGEN]<br>(7/98)						
[4] [2-3, 7]	Cows	2013-<br>2014	98							5%<br>(5/98)	
[4] [2-3, 5]	Calves	2013-<br>2014	98	24%<br>(23/98)	14%[TGEN]<br>(14/98)	8%<br>(8/98)	81%[FLOR]<br>(79/98)	19%<br>(19/98)	55%<br>(54/98)	2%<br>(2/98)	31% [TSDOX]<br>(30/98)
[4] [2-3, 7]	Calves	2013-<br>2014	98							44%<br>(43/98)	
[4] [2-3, 7]	Calves	2013-<br>2014	98							11%[NEOSUL]<br>(11/98)	
[5][2,5]	Cows	2013-<br>2014	100	4%[ERYTH]<br>(4/100)	3%[CCFA]<br>(3/100)		9%[FLOR]<br>(9/100)	4%[BPrP]<br>(4/100)	80%[OXYTET]<br>(80/100)	5%[SMT]<br>(5/100)	7%[TSDOX]<br>(7/100)
[5][2,5]	Cows	2013-<br>2014	100	6%[TILM]<br>(6/100)	1%[CHCl]<br>(1/100)		20%[FLOR+]<br>(20/100)	5% [BPrPB]<br>(5/100)	1%[TETHCl]<br>(1/100)		
[5][2,5]	Cows	2013-<br>2014	100	10%[TULA]<br>(10/100)	5%[CNa]<br>(5/100)			29%[PPG]<br>(29/100)			
[5][2,5]	Calves	2013-	100	2%[GAMI]	6% [CCFA]	8. 0%[ENRO]	28%[FLOR]	6% [BPrP]	54%[OXYTET]	45%[SMT]	30%[TSDOX]

Citation	Population[1]	Sample year	Number	Percentage of herds reporting use (number reporting/total herds)							
		2014		(2/100)	(6/100)	(8/100)	(28/100)	(6/100)	(54/100)	(45/100)	(30/100)
[5] [2,5]	Calves	2013-2014	100	11% [TILM] (11/100)	9%[CNa] (9/100)		63%[FLOR+] (63/100)	14%[PPG] (14/100)	2%[TETHCl] (2/100)		
[5] [2, 5]	Calves	2013-2014	100	12% [TULA] (12/100)							
[6][3, 8]	All	2013-2014	100	44% (44/100)	18%[TGEN] (18/100)	9% (9/100)	81% (81/100)	41% (41/100)	84% (84/100)	34% (34/100)	N/R
[7] [2-3, 8, 12]	All	2007-2008	650	7.4% (48/650)	3.1%[CEFT] (20/650)	3.6% (23/650)	10.4%[FLOR] (68/650)		36.3% (26/650)	7.8% (51/650)	
[7][2-3,9,12]	All	2007-2008	650	0.9% (6/650)					14.6% (95/650)	2.1% (14/650)	
[8][2-4-,5]	All	2002	203	27%[TILM] (54/203)	8.9%[CEFT] (18/203)	0.5%[ENRO] (1/203)	43%[FLOR] (88/203)	38.0% (77/203)	61.0%[TET-OXYTET] (123/203)	59.0% (120/203)	
[8][2-4, 5]	All	2002	203					9.9%[AMP-SUL] (20/203)			
[9][2,8]	All	1999-2002	8	38%[TILM] (3/8)	13%[CEFT] (1/8)	0[ENRO] (0/8)	75%[FLOR] (6/8)	88% (7/8)	88% (7/8)		63% [TSDOX] (5/8)
[9][2,8]	All	1999-2002	8	13%[TYLO] (1/8)				13%[AMPSUL] (1/8)			
[9][2,8]	All	1999-2002	8	0[TYLO] (0/8)					13%[OXYTET] (1/8)		
[9][2,8]	All	1999-2002	8						0[CHLTET]		
[9][2,8]	All	1999-2002	8						13%[CHLTET] (1/8)		

Citation	Population[1]	Sample year	Number	Percentage of herds reporting use (number reporting/total herds)
[9][2,8]	Calves	1999-2002	8	13%[NEOSUL] (1/8)
[9][2,8]	Calves	1999-2002	8	13%[CHLSUL] (1/8)

Notes: Where authors report specific drugs, these are presented in their respective class columns and superscripted with specific drug (abbreviated). An additional superscript will be appended to the citation where specific drug brands are also reported in the paper. Cephalosporins, where only reported to class (as opposed to specific drugs), will be superscripted to indicate which generation if that information is supplied by authors.

Number and percentage of herds reporting usage are extracted from cited papers where this is reported without requiring additional calculations.

[1] Populations refer to those extracted from respective studies, within the scope of this review. Other column data fields, such as herd number and sampling, similarly refer to populations relevant for this review.

Drugs reported: [2] by generic drug [3] by class, [4] by brand.

Administration route: [5] route not specified by authors, [6] pneumatic dart, [7] oral bolus, [8] injectable, [9] in-feed, [10] in-water.

All prevalence usage estimates aggregated by herd; data presented pertains to injectable antimicrobial usage in cow–calf herds investigated in each study. For each study, participants were asked which antimicrobials presented in a booklet or table of choices were used on their farm, with the exception of the following:

[11] Participants were asked to identify the primary antimicrobial used in their herd.

[12] Participants were asked to identify which 5 antimicrobials were used most frequently in their herd.

Abbreviations:

AMP= Ampicillin; AMPSUL = ampicillin–sulbactam; BPrP = benzyl procaine penicillin; BPrPB = benzyl procaine benzathine penicillin; CCFA = ceftiofur crystalline free acid; CEFT = ceftiofur; CEPH = cephapirin benzathine; CHCl = ceftiofur hydrochloride; CNa = ceftiofur sodium; CPRNa = cephapirin sodium; CHLTET = chlortetracycline; CHLSUL = chlortetracycline sulfamethazine; ENRO = enrofloxacin; ERYTH = erythromycin; FLOR = florfenicol; FLOR+ = florfenicol – flunixin meglumine; NEOSUL = neomycin–sulfamethazine; GAMI = gamithromycin; OXYTET = oxytetracycline; PPG = procaine penicillin G; SMT = sulfamethazine; SDOXT = ; SANI = sulfanilamide; SGUA = sulfaguanidine; TET = tetracycline; TETHCL = tetracycline hydrochloride; TILM = tilmicosin; TULA = tulathromycin; TYLO = tylosin; TSDOX = trimethoprim–sulfadoxine; FGEN = authors report cephalosporin class confined to first generation cephalosporin; TGEN = authors report cephalosporin third generation cephalosporin

References

[1] Fossen, J.D.; Campbell, J.R.; Gow, S.P.; Erickson, N.; Waldner, C.L. Antimicrobial Use in Canadian Cow–Calf Herds. Vet. Sci. 2023, 10, 366. https://doi.org/10.3390/vetsci10050366

[2] Ekakoro, J.E., Caldwell, M., Strand, E.B., Strickland, L., Okafor, C.C. A survey of antimicrobial use practices of Tennessee beef producers. *BMC Vet. Res*. 2019, 15, 222-233. DOI: 10.1186/s12917-019-1978-6

[3] Beef cow-calf health and management practices in the United States, 2017, report 2, USDA. Available online: https://www.aphis.usda.gov/animal_health/nahms/beefcowcalf/downloads/beef2017/beef-2017-part2.pdf (accessed on 27 May, 2022).

[4] Waldner, C. L., Gow, S., Parker, S., Campbell, J. R. Antimicrobial resistance in fecal *Escherichia coli* and *Campylobacter* spp. from beef cows in western Canada and associations with herd attributes and antimicrobial use. *Can. J. Vet. Res.* 2019, 83, 80-89.

[5] Waldner, C.L., Parker, S., Gow, S., Wilson, D.J., Campbell, J.R. Antimicrobial usage in western Canadian cow-calf herds. *Can. Vet. J.* 2019, 60, 255–267.

[6] Waldner, C.L., Parker, S., Gow, S., Wilson, D.J., Campbell, J.R. Attitudes towards antimicrobial use and factors associated with antimicrobial use in western Canadian cow-calf herds. *Can. Vet. J***.** 2019, 60, 391–398.

[7] Green, A.L., Carpenter, L.R., Edmisson, D.E., Lane, C.D., Welborn, M.G., Hopkins, F.M., Bemis, D.A., Dunn, J.R. Producer attitudes and practices related to antimicrobial use in beef cattle in Tennessee. *J. Am. Vet. Med. Assoc*. 2010, 237, 1292-1298. https://doi.org/10.2460/javma.237.11.1292

[8] Gow, S.P., Waldner, C.L. Antimicrobial drug use and reason for treatment in 203 western Canadian cow–calf herds during calving season. *Prev. Vet. Med.* 2009, 90, 55-65. DOI:10.1016/j.prevetmed.2009.03.010.

[9] Gow, S.P., Waldner, C.L., Rajic, A., McFall, M.E., Reid-Smith, R. Prevalence of antimicrobial resistance in fecal generic *Escherichia coli* isolated in western Canadian beef herds. Part II — Cows and cow-calf pairs. *Can. J. Vet. Res.* 2008, 72, 91-100.

Table S2. Risk factors for antimicrobial usage reported in 7 included studies in beef cow–calf herds in the United States and Canada

Broad category	Citation	Specific exposure	Specific outcome	OR	95% CIs	*P* value
			**Herd-level predictors**			
**Producer attributes**						
		**Age**				
	[1]	At least 1 decision-maker < 30 y	30% of either cows or calves received treatment with antimicrobials	1.31	(0.20, 8.51)	0.78
	[1]	At least 1 decision-maker < 30 y	Antimicrobials used in the feed or water	1.52	(0.64, 3.56)	0.34
	[1]	At least 1 decision-maker < 30 y	Medically important antimicrobials used for disease prevention in cows or pre-weaned calves	1.21	(0.19, 7.18)	0.85
	[1]	At least 1 decision-maker < 30 y	Any Health Canada Category I drugs used in the herd	2.09	(1.1, 3.97)	0.02
	[1]	At least 1 decision-maker < 30 y	Number of reasons for use and production groups where Health Canada Category I drugs were used in the herd	2.49	(1.07, 5.79)	0.04
**Herd attributes**		**Herd size**				
	[2]	Every additional 50 cows	Odds of having had to treat >10% of calves at least once in the past 3 y	Decreased 1.28	(1.06, 1.54)	0.01
	[3]	Herd size > 50	AMU- all drugs	2.4	(1.6, 3.8)	<0.01
	[4]	Herd size	Degree of concern regarding AMR			0.393
	[1]	Number of cows	30% of either cows or calves received treatment	1.47	(0.37, 5.83)	0.58

Broad category	Citation	Specific exposure	Specific outcome	OR	95% CIs	*P* value
		calving > 500	with antimicrobials			
	[1]	Number of cows calving > 500	Antimicrobials used in the feed or water	1.08	(0.47, 2.5)	0.86
	[1]	Number of cows calving > 500	Medically important antimicrobials used for disease prevention in cows or pre-weaned calves	0.62	(0.09, 4.35)	0.63
	[1]	Number of cows calving > 500	Any Health Canada Category I drugs used in the herd	0.78	(0.28, 2.15)	0.64
	[1]	Number of cows calving > 500	Number of reasons for use and production groups where Health Canada Category I drugs were used in the herd	0.72	(0.19, 2.74)	0.64
	[5]	Herds with > 300 cows	Treat > 5% of calves with AMs for diarrhea			0.30
	[5]	Herds with > 300 cows	Treat > 5% of calves with AMs for respiratory disease	RR = 1.68	(0.91, 3.3)	0.10
	[5]	Herds with > 300 cows	Treat > 5% of cows for lameness			0.67
	[2]	Every additional 50 cows in herd size	Decreased odds of having to treat > 10% of calves for scours	1.28	(1.06, 1.54)	0.01
**Type of operation**	[3]	MOT greater odds usage relative to cow–calf	Tetracycline usage via injectable or oral route during past year			0.16
	[3]	MOT greater odds usage relative to cow–calf	Beta Lactam usage via injectable or oral route during past year			0.11
	[3]	MOT greater odds usage relative to cow–calf	Florfenicol usage via injectable or oral route during past year			<0.01
	[3]	MOT greater odds	Macrolide usage via injectable or oral route dur-			<0.01

Broad category	Citation	Specific exposure	Specific outcome	OR	95% CIs	*P* value
		usage relative to cow–calf	ing past year			
	[3]	MOT greater odds usage relative to cow–calf	Sulfonamide usage via injectable or oral route during past year			0.04
	[3]	MOT greater odds usage relative to cow–calf	Fluoroquinolone usage via injectable or oral route during past year			0.07
	[3]	MOT greater odds usage relative to cow–calf	Ceftiofur usage via injectable or oral route during past year			0.04
	[3]	MOT greater odds usage relative to cow–calf	Aminoglycoside usage via injectable or oral route during past year			0.01
	[3]	MOT greater odds usage relative to cow–calf	Lincomycin usage via injectable or oral route during past year			0.99
	[3]	MOT greater odds usage relative to cow–calf	Aminocyclitols usage via injectable or oral route during past year			0.93
	[3]	MOT greater odds usage relative to cow–calf	Any AM usage via injectable or oral route during past year			<0.01
	[3]	MOT greater odds usage relative to cow–calf	Tetracyclines administered in feed in past year			0.16
	[3]	MOT greater odds usage relative to cow–calf	Sulfonamide administered in feed in past year			<0.01

Broad category	Citation	Specific exposure	Specific outcome	OR	95% CIs	*P* value
	[3]	MOT greater odds usage relative to cow–calf	Beta Lactams administered in feed in past year			<0.01
	[3]	MOT greater odds usage relative to cow–calf	Macrolide administered in feed in past year			0.41
	[3]	MOT greater odds usage relative to cow–calf	Aminoglycoside administered in feed in past year			0.30
	[3]	MOT greater odds usage relative to cow–calf	Any AM administered in feed in past year			<0.01
**Vaccines**		**Scours**				
	[2]	Scours vaccination	Proportion calves treated for scours			0.19
	[2]	Scours vaccination	Proportion calves treated for scours			0.27
		**Clostridial vaccines**				
	[2]	Herds vaccinate cows in fall for clostridial disease	Number of calves treated for scours in spring	1.85	(1.2, 2.87)	0.01
	[2]	Herds do not vaccinate cows for clostridial disease	Number of calves treated for scours in spring	1.54	(1.04, 2.21)	0.03
**Calving season**		**Start date**				
	[2]	Every 1 mo delay in the start of the calving season	Odds of having had to treat ≥ 10% of calves at least once in the past 3 y for scours	Decreased 1.4	(1.07, 1.83)	0.015

Broad category	Citation	Specific exposure	Specific outcome	OR	95% CIs	*P* value
	[2]	Every 1 mo delay in the start of the calving season	Odds of having had to treat ≥10% of calves at least once in the past 3 y for pneumonia	Decreased 1.79	(1.5, 2.14)	
	[1]	Calving started before March 2014	30% of either cows or calves received treatment with antimicrobials	2.3	(0.81, 6.57)	0.12
	[1]	Calving started before March 2014	Antimicrobials used in the feed or water	1.01	(0.58, 1.76)	0.98
	[1]	Calving started before March 2014	Medically important antimicrobials used for disease prevention in cows or pre-weaned calves	1.92	(0.72, 5.14)	0.20
	[1]	Calving started before March 2014	Any Health Canada Category I drugs used in the herd	1.28	(0.74, 2.21)	0.38
	[1]	Calving started before March 2014	Number of reasons for use and production groups where Health Canada Category I drugs were used in the herd	1.12	(0.51, 2.46)	0.77
	[5]	Calving started before March	Treat > 5% of calves with AMs for diarrhea			0.26
	[5]	Calving started before March	Treat > 5% of calves with AMs for respiratory disease	RR =1.70	(0.91, 3.17)	0.09
	[5]	Calving started before March	Treat > 5% of cows for lameness			0.84
	[2]	Every 1 mo delay in the start of calving season	Decreased odds of having had to treat > 10% of calves at least once in the past 3 y	1.40	(1.07, 1.83)	0.015
	[2]	Every 1 mo delay in the start of calving season	Decreased odds of having had to treat > 10% of calves at least once in the past 3 y for pneumonia	1.79	(1.50, 2.14)	
**Use of AI**	[6]	Herd use of AI for breeding some of the	Increased odds of treating >5% of nursing calves for respiratory disease	N/R		< 0.05

Broad category	Citation	Specific exposure	Specific outcome	OR	95% CIs	*P* value
		herd cows				
**Herd health outcome measures**		**Calf mortality**				
	[1]	Calf mortality in 2013 > 5%	30% of either cows or calves received treatment with antimicrobials	1.88	(0.67, 5.23)	0.23
	[1]	Calf mortality in 2013 > 5%	Antimicrobials used in the feed or water	1.64	(0.95, 2.81)	0.07
	[1]	Calf mortality in 2013 > 5%	Medically important antimicrobials used for disease prevention in cows or pre-weaned calves	0.45	(0.06, 3.25)	0.43
	[1]	Calf mortality in 2013 > 5%	Any Health Canada Category I drugs used in the herd	1.43	(0.06, 3.25)	0.21
	[1]	Calf mortality in 2013 > 5%	Number of reasons for use and production groups where Health Canada Category I drugs were used in the herd	1.87	(0.84, 4.14)	0.13
	[7]	% cow treated in herd	Calf treatment	1.2	(1.1, 1.3)	
	[7]	% heifers treated in herd	Calf treatment	0.2	(0.2, 0.3)	
**Herd management**						
		**Biosecurity and usage policies**				
	[3]	BQA certification	AMU- all	1.7	(1.2, 2.5)	<0.01

Broad category	Citation	Specific exposure	Specific outcome	OR	95% CIs	*P* value
	[3]	Quarantine newly purchased animals	AMU- all	1.6	(1.1, 2.2)	<0.01
	[3]	Written instructions for treating disease	AMU- all	1.9	(1.1, 3.0)	<0.01
	[3]	Observed withdrawal times	AMU- all	2.1	(1.4, 3.5)	<0.01
**Marketing**		**Retained ownership**				
	[1]	Calves retained after weaning	30% of either cows or calves received treatment with antimicrobials	1.99	(0.72, 5.49)	0.19
	[1]	Calves retained after weaning	Antimicrobials used in the feed or water	1.51	(0.88, 2.6)	0.13
	[1]	Calves retained after weaning	Medically important antimicrobials used for disease prevention in cows or pre-weaned calves	1.7	(0.65, 4.49)	0.28
	[1]	Calves retained after weaning	Any Health Canada Category I drugs used in the herd	1.92	(1.12, 3.29)	0.02
	[1]	Calves retained after weaning	Number of reasons for use and production groups where Health Canada Category I drugs were used in the herd	2.06	(0.94, 4.52)	0.07
	[5]	Produce purebred cattle	Treat > 5% of calves with AMs for diarrhea			0.61
	[5]	Produce purebred cattle	Treat > 5% of calves with AMs for respiratory disease			0.40
	[5]	Produce purebred cattle	Treat > 5% of cows for lameness			0.81
			**<u>Individual-level predictors</u>**			
**Calving**		**Dystocia/post-**				

Broad category	Citation	Specific exposure	Specific outcome	OR	95% CIs	*P* value
		**partum illness**				
	[7]	Cows receiving manipulation/traction/c-s	Cow treatment	2.1	(1.7, 2.7)	
	[7]	Heifers receiving manipulation/traction/c-s	Heifer treatment	16	(8.8, 24)	
	[7]	Cows with post-calving problems	Cow treatment	59	(27, 110)	
	[7]	Heifer with post-calving problems	Heifer treatment	312	(50,1941)	
**Class of livestock**						
	[7]	Heifers vs. other breeding cows		0.2	(0.2, 0.3)	
**Sex**	[7]	Female calf vs male calf	Calf treatment	0.8	(0.7, 0.9)	
**Calving**						
	[7]	Calves involved in manipulation or traction	Calf treatment	1.5	(1.3, 1.7)	

Abbreviations:

95% CIs = 95% confidence intervals; AM = antimicrobial; AMU = antimicrobial use; AMR = antimicrobial resistance; BQA = Beef Quality Assurance; C-S = caesarean section; Mo = month; MOT = mixed operation type (feedlot + cow–calf); OR = odds ratio; RR = relative risk; Y = years.

References

[1] Waldner, C.L., Parker, S., Gow, S., Wilson, D.J., Campbell, J.R. Attitudes towards antimicrobial use and factors associated with antimicrobial use in western Canadian cow-calf herds. *Can. Vet. J.* 2019, 60, 391–398.
[2] Waldner, C., Jelinski, M.D., McIntyre-Zimmer, K. Survey of western Canadian beef producers regarding calf-hood diseases, management practices, and veterinary service usage. *Can. Vet. J.* 2013, 54, 559–564.
[3] Green, A.L., Carpenter, L.R., Edmisson, D.E., Lane, C.D., Welborn, M.G., Hopkins, F.M., Bemis, D.A., Dunn, J.R. Producer attitudes and practices related to antimicrobial use in beef cattle in Tennessee. *J. Am. Vet. Med. Assoc.* 2010, 237, 1292-1298. https://doi.org/10.2460/javma.237.11.1292
[4] Ekakoro, J.E., Caldwell, M., Strand, E.B., Strickland, L., Okafor, C.C. A survey of antimicrobial use practices of Tennessee beef producers. *BMC Vet. Res.* 2019, 15, 222-233. DOI: 10.1186/s12917-019-1978-6
[5] Waldner, C.L., Parker, S., Gow, S., Wilson, D.J., Campbell, J.R. Antimicrobial usage in western Canadian cow-calf herds. *Can. Vet. J.* 2019**,** 60, 255–267.
[6] Fossen, J.D., Campbell, J.R., Gow, S.P., Erickson, N., Waldner, C.L. Antimicrobial Use in Canadian Cow–Calf Herds. *Vet. Sci*. 2023;10:366. doi: https://doi.org/10.3390/vetsci10050366.
[7] Gow, S.P., Waldner, C.L. Antimicrobial drug use and reason for treatment in 203 western Canadian cow–calf herds during calving season. *Prev. Vet. Med.* 2009, 90, 55-65. DOI:10.1016/j.prevetmed.2009.03.010.

Table S3. Risk factors associated with antimicrobial resistance reported in 7 included studies in beef cow–calf herds in the United States and Canada

Broad category	Specific category	Citation	Specific exposure	Specific outcome	OR	95% CIs	*P* value
			**Herd level predictors**				
**Herd attributes**		[1]	Producer age	Resistance to ≥ 1 AM	1.0	(0.96, 1.1)	0.60
			Produces purebred cattle	Resistance to ≥ 1 AM	1.5	(0.23, 7.4)	0.82
		[1]	Calves retained after weaning	Resistance to ≥ 1 AM	0.04	(0.06, 3.7)	0.86
		[1]	Calving begins prior to March in 2014	Resistance to ≥ 1 AM	1.6	(0.34, 7.5)	0.70
		[2]	Location: CA vs. WA-OR	Resistance to > 1 AM	2.23	(1.33, 3.74)	< 0.01
			Location: dairy-intense vs. remote from dairies	Resistance to > 1 AM	2.8	(1.16, 6.74)	0.02
		[3]	Large herds relative to medium/small herds	Resistance to CRB	1.58	(1.17, 2.14)	0.003
**Herd health**							
	Calf mortality	[1]	Herds with ≥ 5% preweaning calf mortality	≥ 1 *E. coli* isolate with resistance to streptomycin and sulfisoxazole	7.8	(1.2, 61)	0.03
		[1]	Herds with ≥5% preweaning calf mortality	Resistance to 1 or more AMs	3.8	(0.68, 19)	0.14
		[1]	Herds with ≥ 5% preweaning calf mortality	Resistance to 2 or more AMs	5.8	(0.94, 36)	0.16

Broad category	Specific category	Citation	Specific exposure	Specific outcome	OR	95% CIs	*P* value
	Biosecurity						
		[3]	Number of times water trough cleaned per month	Cefotaxime resistant bacterial prevalence	0.218		0.001
		[3]	Cleaning product used bleach (Referent = none)	Cefotaxime resistant bacterial prevalence	0.324		<0.001
		[3]	Dead disposal (Referent = bury)	Cefotaxime resistant bacterial prevalence	1.94		<0.001
		[3]	Isolate/Quarantine new arrivals (Referent = No)	Cefotaxime resistant bacterial prevalence	0.391		<0.001
**Herd structure/demographic**							
	Classes of cattle						
		[4]	Calves relative to cows	Sulfamethoxazole resistance in *E. coli*	3.7	(1.11, 12.34)	
		[4]	Calves relative to cows	Streptomycin resistance in *E. coli*			Non-sig
		[4]	Calves relative to cows	Tetracycline resistance in *E. coli*			Non-sig
		[5]	Calves relative to cows	≥ 1 resistant isolate in herd	10	(5.8, 17.0)	< .0001

Broad category	Specific category	Citation	Specific exposure	Specific outcome	OR	95% CIs	*P* value
				in spring			
		[5]	Calves relative to cows	≥ 1 resistant isolate in herd in fall	1.1	(0.3, 3.7)	0.91
		[2]	Calves relative to cows	Resistance to > 1 AM	2.33	(0.98, 5.56)	0.06
		[5]	Cows in herd with 1 isolate resistant to sulfamethoxazole-spring	Calves in herd with 1 isolate resistant to sulfamethoxazole in spring	7.5	(1.3, 41.7)	0.02
		[5]	Cows in herd with 1 isolate resistant to tetracyclines-spring	Calves in herd with 1 isolate resistant to tetracyclines in spring	6.1	(1.5, 25.3)	0.01
		[5]	Dam resistant to at least one AM	Calf resistant to at least one AM	N/R	N/R	0.36
	Herd size						
		[3]	Total number cows	Cefotaxime resistant bacterial prevalence	0.99		0.19
		[3]	Herd size categorized: medium-small referent	Cefotaxime resistant bacterial prevalence	1.58		0.003
		[1]	Total cows calved in 2014 > 300	Resistance to ≥ 1 AM	0.22	(0.005, 1.7)	0.23
		[1]	Number of cows to calves in 2014	Resistance to ≥ 1 AM	0.996	(0.989, 1.0)	0.17
	Farm area in acres	[3]	Farm size in acres	Cefotaxime resistant bacterial prevalence	0.999		0.248
	Sample collection						
		[1]	Samples collected [from cows] after weaning	Resistance to ≥ 1 AM in herd	0.85	(0.17, 5.5)	0.99

Broad category	Specific category	Citation	Specific exposure	Specific outcome	OR	95% CIs	*P* value
**AMU**							
		[1]	AM provided to cows in feed	Resistance to ≥ 1 AM in herd	2.9	(0.42, 15)	0.32
		[1]	Injectable AMs used in cows	Resistance to ≥ 1 AM in herd	0.98	(0.13, ∞)	0.99
		[1]	Highest % of cows in which AMs were used for any reason	Resistance to ≥ 1 AM in herd	1.0	(0.99, 1.0)	0.31
	Class tx						
	Aminoglycosides (not topical)	[1]	Aminoglycosides (not topical)	Resistance to ≥ 1 AM in herd	8.6	(0, 335)	0.99
	1st gen cephalosporins	[1]	1st gen cephalosporins	Resistance to ≥ 1 AM in herd	2.2	(0, 22)	0.99
	Ceftiofur	[1]	Ceftiofur	Resistance to ≥ 1 AM in herd	1.0	(0, 7.9)	0.99
		[6]	Ceftiofur tx in cows	Phenotypical cephalosporin-resistant *E. coli*			Non-sig
		[6]	Ceftiofur tx in cows	Phenotypical tetracycline-resistant *E. coli*			Non-sig
		[6]	Ceftiofur tx in cows	Phenotypical TMS -resistant *E. coli*			Non-sig
		[6]	Ceftiofur tx in cows	Phenotypical nalidixic acid -resistant *Salmonella*			Non-sig
		[6]	Ceftiofur tx in cows	Phenotypical cephalospor-			Non-

Broad category	Specific category	Citation	Specific exposure	Specific outcome	OR	95% CIs	*P* value
				in-resistant *Salmonella*			sig
	Florfenicol	[1]	Herds in which cows were treated with florfenicol	*E. coli* resistance to ≥ 2 antimicrobials	7.1	(1.1, 57)	0.03
		[1]	Herds in which cows were treated with florfenicol	Resistance to ≥ 1 AM in herd	2.9	(0.60, 14)	0.22
	Ionophore feeding	[3]	Ionophore feeding (Referent = no)	Cefotaxime resistant bacterial prevalence	0.742		0.042
		[1]	Lasalocid feeding cows	Resistance to ≥ 1 AM in herd	9.0	(0.11, 750)	0.40
		[1]	Monensin feeding cows	Resistance to ≥ 1 AM in herd	2.8	(0.24, 19)	0.47
		[7]	Proportion of herd isolates positive for resistance in spring	Proportion of herd isolates positive for resistance in fall			0.82
		[7]	Proportion of calves positive for resistance in spring	Proportion of calves positive for resistance in fall			0.82
	Macrolide	[1]	Herds using macrolides in cows	Resistance to ≥ 1 AM in herd	0.30	(0, 2.0)	0.26
	Penicillin	[1]	Herds using penicillins in cows	Resistance to ≥ 1 AM in herd	1.9	(0.39, 8.8)	0.54
	Polymyxins	[1]	Herds using polymyxins in cows	Resistance to ≥ 1 AM in herd	0.46	(0, 3.2)	0.49
	Sulphonamides	[1]	Herds using sulfa boluses including intra-uterine	Resistance to ≥ 1 AM in herd	2.3	(0.04, 26)	0.86

Broad category	Specific category	Citation	Specific exposure	Specific outcome	OR	95% CIs	*P* value
	Tetracyclines	[1]	Herds using tetracyclines in cows	Resistance to ≥ 1 AM in herd	0.60	(0.13, 4.1)	0.76
	Trimethoprim-sulfadoxine	[1]	Herds using trimethoprim–sulfadoxine in cows	Resistance to ≥ 1 AM in herd	1.5	(0.03, 15)	0.99
	MIA used	[1]	Cat 1 drugs used in cows	Resistance to ≥ 1 AM in herd	0.26	(0, 1.8)	0.19
			Cat 2 drugs used in cows	Resistance to ≥ 1 AM in herd	1.0	(0.21, 4.7)	0.99
**Season**	Spring relative to fall	[7]	Spring relative to fall	1+ resistant isolates in herd	9.6	(4.5, 20.7)	
		[7]	Proportion of isolates positive for resistance in the spring	Proportion of isolates positive for resistance in the fall			0.82
		[7]	Proportion of calves positive for resistance in the spring	Proportion of calves positive for resistance in the fall			0.37
			**Individual level predictors**				
**Demographics**							
	Sex	[7]	Calf sex association in spring	Calf AMR status			0.54
		[7]	Calf sex association in	Calf AMR status			0.85

Broad category	Specific category	Citation	Specific exposure	Specific outcome	OR	95% CIs	*P* value
			fall				
	Age	[7]	Calf age: < 3 d relative to > 10 d association in spring	Calf AMR status	0.55	(0.3, 1.0)	0.03
		[7]	Calf age: < 3 d relative to > 10 d association in fall	Calf AMR status			0.75
	Breed	[7]	Calf breed: association in spring	Calf AMR status			0.40
			Calf breed: association in fall	Calf AMR status			0.38
	Dam	[5]	Dam resistant to 1+ AMs	Calf resistant to 1+ AMs			0.36
**Calving**			Dam age	Calf AMR status			0.72
	Post-calving illness	[7]	Classification as “unhealthy”	Calf AMR status	4.3	(1.16, 2.04)	0.0001
**AMU**							
	Previous treatment						
		[5]	Dam resistant to at least one AM	Calf resistant to at least one AM	N/R	N/R	0.36
		[5]	Calf prior tx: association in spring	Calf AMR status			0.65

Broad category	Specific category	Citation	Specific exposure	Specific outcome	OR	95% CIs	*P* value
		[5]	Calf prior tx: association in fall	Calf AMR status			0.13
		[5]	Number of days from last tx association in spring	Calf AMR status			0.92
		[5]	Number of days from last tx: association in spring	Calf AMR status			0.74

Abbreviations:

95% CIs = 95% confidence intervals; AM = antimicrobial; AMU = antimicrobial use; CRB = cefotaxime-resistant bacteria; Cat = category; D = days; *E. coli* = *Escherichia coli*; Gen = generation; MIA = medically important antimicrobial; Non-sig = non-significant; N/R = not reported; OR = ODDS RATIO; TMS = trimethoprim–sulfa; Tx = treatment.

References

[1] Waldner, C.L., Gow, S., Parker, S., Campbell, J.R. Antimicrobial resistance in fecal *Escherichia coli* and *Campylobacter* spp. from beef cows in western Canada and associations with herd attributes and antimicrobial use. *Can. J. Vet. Res.* 2019, 83, 80-89.

[2] Berge, A.C., Hancock, D.D., Sischo, W.M., Besser, T.E.. Geographic, farm, and animal factors associated with multiple antimicrobial resistance in fecal *Escherichia coli* isolates from cattle in the western United States. J. Am. Vet. Med. Assoc. 2010; 236: 1338-1344.

[3] Markland, S., Weppelmann,T.A., Ma, Z., Lee, S., Mir, R.A., Teng, L., Ginn, A., Lee, C., Ukhanova, M., Galindo, S., Carr, C., DiLorenzo, N., Ahn, S., Mah, J-H, Kim, H-Y, Mai, V., Mobley, R., Morris, J.G., Jeong, K.C.C. High prevalence of cefotaxime resistant bacteria in grazing beef cattle: a cross sectional study. *Front. Microbiol*. 2019, 10, 176 DOI:https://doi.org/10.3389/fmicb.2019.00176.

[4] Carson, C.A., Reid-Smith, R., Irwin, R.J., Martin, W.S., McEwen, S.A. Antimicrobial use on 24 beef farms in Ontario. *Can. J. Vet. Res.* 2008, 72,109-118.

[5] Gow, S.P., Waldner, C.L., Rajic, A., McFall, M.E., Reid-Smith, R. Prevalence of antimicrobial resistance in fecal generic *Escherichia coli* isolated in western Canadian beef herds. Part II — Cows and cow-calf pairs. *Can. J. Vet. Res.* 2008, 72, 91-100.

[6] Agga, G.E., Schmidt, J.W., Arthur, T.A. Antimicrobial-resistant fecal bacteria from ceftiofur-treated and nonantimicrobial-treated comingled beef cows at a cow–calf operation. *Microb. Drug Res.* 2016, 2, 598 - 608. https://doi-org.cyber.usask.ca/10.1089/mdr.2015.0259S

[7] Gow, S.P., Waldner, C.L., Rajic, A., McFall, M.E., Reid-Smith, R. Prevalence of antimicrobial resistance in fecal generic *Escherichia coli* isolated in western Canadian cow-calf herds. Part I — Beef calves. Can. J. Vet. Res. 2008, 72, 82-90.

Table S4. Descriptive summary of antimicrobial resistance of enteric bacteria reported in 6 included studies of antimicrobial resistance in beef cow–calf herds in the United States and Canada

Citation	Population[1]	Bacterial species	Number isolates	Level of Aggregation	Resistance to ≥ 1 Antimicrobial	Resistance Cephalosporins	Resistance Tetracyclines	Resistance Diaminopyrimidines-sulfonamides	Resistance Aminoglycosides	Resistance Sulfonamides
[1]	Cows	*E. coli*	305	Isolate	3.9% (12/305)	0 CEFT	3.3% (10/305)	0.3% TMSX (1/305)	2.6% STREP (8/305)	2.6% SULF (8/305)
[1]	Cows	*E. coli*	305	Isolate		0 CEFR				
[1]	Cows	*E. coli*	305	Isolate		0 CEFX				
[1]	Cows	*E. coli*	105	Herd	9.5% (10/105)	0 CEFT	7.6% (8/105)	1.0% TMSX (1/105)	7.4% STREP (7/105)	7.4% SULF (7/105)
[1]	Cows	*E. coli*	105	Herd		0 CEFR				
[1]	Cows	*E. coli*	105	Herd		0 CEFX				
[1]	Cows	*Campylobacter spp.*	87	Isolate	18.0% (16/87)	N/R	15.0% (13/87)		0 GENT	
[2]	Cows	CRB	457	Isolate		41.8% CEFO (217/457)				
[2]	Calves	CRB	383	Isolate		54.0% CEFO (207/383)				
[2]	Fecal	CRB	840	Isolate		95.7% CEFO (804/840)				
[2]	Forage	CRB	93	Isolate		96.2% CEFO (92/93)				

Citation	Population[1]	Bacterial species	Number isolates	Level of Aggregation	Resistance to ≥ 1 Antimicrobial	Resistance Cephalosporins	Resistance Tetracyclines	Resistance Diaminopyrimidines-sulfonamides	Resistance Aminoglycosides	Resistance Sulfonamides
[2]	Soil	CRB	77	Isolate		88.6% ^{CEFO} (62/77)				
[2]	Water	CRB	88	Isolate		88.6% ^{CEFO} (78/88)				
[3]	Cows	*Salmonella spp.*	34	Isolate	0	0^{CEFX}	0	0^{TMSX}	0^{GENT}	0^{SUMT}
[3]	Cows	*Salmonella spp.*	34	Isolate	0	0^{CEFT}			0^{STREP}	
[3]	Cows	*Salmonella spp.*	34	Isolate	0	0^{CEFR}				
[3]	Cows	*Campylobacter spp.*	244	Isolate	43.8% (107/ 244)	N/R	38.9% (94/244)	N/R	0^{GENT}	N/R
[3]	Cows	*E. coli*	1146	Isolate	16.6% (190/1146)	0.2%^{CEFX} (2/1146)	16% (183/1146)	0^{TMSX}	6.5%^{STREP} (74/1146)	6.7%^{SUMT} (77/1146)
[3]	Cows	*E. coli*	1146	Isolate		0.2%^{CEFX} (2/1146)			0.3%^{GENT}	
[4]	Calves	*E. coli*	106	Isolate	88.7% (94/106)	6.6%^{CEFX} (7/106)	86.8% (92/106)	29.2%^{TMSX} (31/106)	1.9%^{GENT} (2/106)	82.1%^{SUMT} (87/106)
[4]	Calves	*E. coli*	106	Isolate		1. 9%^{CEFT} (2/106)			28. 3%^{KANA} (30 /106)	
[4]	Calves	*E. coli*	106	Isolate		0^{CEFR}			6.7%^{STREP}	

Citation	Population[1]	Bacterial species	Number isolates	Level of Aggregation	Resistance to ≥ 1 Antimicrobial	Resistance Cephalosporins	Resistance Tetracyclines	Resistance Diaminopyrimidines-sulfonamides	Resistance Aminoglycosides	Resistance Sulfonamides
									(7/106)	
[4]	Calves	*E. coli*	106	Isolate		7.5%^{CEPH} (8/106)				
[5]	Cows	*E. coli*	602	Isolate	11% (64/602)	N/R	N/R	N/R	N/R	N/R
[5]	Calves	*E. coli*	171	Isolate	19% (33/171)	N/R	N/R	N/R	N/R	N/R
[6]	Cows	*E. coli*	1555	Isolate	9.8% (152/1555)	0.2%^{CEFX} (3/1555)	8.7% (135/1555)	0.5%^{TMSX} (8/1555)	0.2%^{GENT} (3/1555)	7.1%^{SUMT} (110/1555)
[6]	Cows	*E. coli*	1555	Isolate		0.1%^{CEFT} (2/1555)			3.4%^{STREP} (53/1555)	
[6]	Cows	*E. coli*	1555	Isolate		0^{CEFR}				
[6]	Cows	*E. coli*	1555	Isolate		0.3%^{CEPH} (5/1555)				
[6]	Cows	*E. coli*	312	Isolate	6.1% (19/312)	0^{CEFT}	5.1% (16/312)	1.7%^{TMSX} (5/312)	0^{GENT}	4.4%^{SUMT} (14/312)
[6]	Cows	*E. coli*	312	Isolate		0^{CEFX}			3.4%^{STREP} (11/312)	
[6]	Cows	*E. coli*	312	Isolate		0.7%^{CEPH} (2/312)				
[6]	Calves	*E. coli*	318	Isolate	25.8%	2.9%^{CEFX}	20.6%	7.3%^{TMSX}	0.3%^{GENT}	24.3%^{SUMT}

Citation	Population[1]	Bacterial species	Number isolates	Level of Aggregation	Resistance to ≥ 1 Antimicrobial	Resistance Cephalosporins	Resistance Tetracyclines	Resistance Diaminopyrimidines-sulfonamides	Resistance Aminoglycosides	Resistance Sulfonamides
					(82/318)	(9/318)	(66/318)	(23/318)	(1/318)	(77/318)
[6]	Calves	*E. coli*	318	Isolate		0^{CEFT}			13%^{STREP} (41/318)	
[6]	Calves	*E. coli*	318	Isolate		6.0%^{CEFR} (19/318)				
[6]	Calves	*E. coli*	318	Isolate		7.9%^{CEPH} (25/318)				
[6]	Cows	*E. coli*	533	Ind	15.1% (80/533)	0.6%^{CEFX} 6(/533)	13.4% (71/533)	1.7% ^{TMSX} (9/533)	0.4%^{GENT} (2/533)	11.0% ^{SUMT} (59/533)
[6]	Cows	*E. coli*	533	Ind		0.2%^{CEFT} (1/533)			6.7% ^{STREP} (36/533)	
[6]	Cows	*E. coli*	533	Ind		0^{CEFR}				
[6]	Cows	*E. coli*	533	Ind		0.8%^{CEPH} (4/533)				
[6]	Cows	*E. coli*	105	Ind	8.6% (9/105)	0^{CEFX}	7.7% (9/105)	2.0% ^{TMSX} (2/105)	0^{GENT}	5.7%^{SUMT} (6/105)
[6]	Cows	*E. coli*	105	Ind		0^{CEFT}			4.8%^{STREP} (5/105)	
[6]	Cows	*E. coli*	105	Ind		0^{CEFR}				
[6]	Cows	*E. coli*	105	Ind		0.9%^{CEPH} (5/533)				

Citation	Population[1]	Bacterial species	Number isolates	Level of Aggregation	Resistance to ≥ 1 Antimicrobial	Resistance Cephalosporins	Resistance Tetracyclines	Resistance Diaminopyrimidines-sulfonamides	Resistance Aminoglycosides	Resistance Sulfonamides
[6]	Calves	*E. coli*	105	Ind	37.9% (40/105)	4.9%[CEFX] (5/105)	31.6% (33/105)	9.2% [TMSX] (10/105)	0.9%[GENT] (1/105)	33.8% [SUMT] (35/105)
[6]	Calves	*E. coli*	105	Ind		3.8%[CEFT] (4/105)			7.7% [STREP] (9/105)	
[6]	Calves	*E. coli*	105	Ind		0[CEFR]				
[6]	Calves	*E. coli*	105	Ind		10.5%[CEPH] (10/105)				
[6]	Cows	*E. coli*	69	Herd	60.9% (42/69)	4.4%[CEFX] (3/69)	53.6% (37/69)	8.7% [TMSX] (6/69)	2.9%[GENT] (2/69)	49.3% [SUMT] (34/69)
[6]	Cows	*E. coli*	69	Herd		1.5%[CEFT] (1/69)			34.8%[STREP] (24/69)	
[6]	Cows	*E. coli*	69	Herd		0[CEFR]				
[6]	Cows	*E. coli*	69	Herd		5.8%[CEPH] (4/69)				
[6]	Cows	*E. coli*	10	Herd	60% (6/10)	0[CEFX]	50% (5/10)	10% [TMSX] (1/10)	0[GENT]	50% [SUMT] (5/10)
[6]	Cows	*E. coli*	10	Herd		0[CEFT]			40%[STREP]	

Citation	Population[1]	Bacterial species	Number isolates	Level of Aggregation	Resistance to ≥ 1 Antimicrobial	Resistance Cephalosporins	Resistance Tetracyclines	Resistance Diaminopyrimidines-sulfonamides	Resistance Aminoglycosides	Resistance Sulfonamides
									(4/10)	
[6]	Cows	*E. coli*	10	Herd		0[CEFR]				
[6]	Cows	*E. coli*	10	Herd		10%[CEPH] (1/10)				
[6]	Calves	*E. coli*	10	Herd	100% (10/10)	30%[CEFX]	100% (10/10)	40% [TMSX] (4/10)	10%[GENT] (1/10)	100%[SUMT] (10/10)
[6]	Calves	*E. coli*	10	Herd		20%[CEFT] (2/10)			80%[STREP] (8/10)	
[6]	Calves	*E. coli*	10	Herd		0[CEFR]				
[6]	Calves	*E. coli*	10	Herd		70%[CEPH] (7/10)				

Notes: Where authors report specific drugs, these are presented in their respective class columns and superscripted with specific drug (abbreviated). An additional superscript will be appended to the citation where specific drug brands are also reported in the paper. Cephalosporins, where only reported to class (as opposed to specific drugs), will be superscripted to indicate which generation if that information is supplied by authors.

The frequency of usage is extracted from cited papers where this is reported without requiring additional calculations.

[1] Populations refer to those extracted from respective studies, within the scope of this review. Other column data fields, such as herd number and sampling, similarly refer to populations relevant to this review.

Abbreviations:

CRB = cefotaxime resistant bacteria; CEFT = ceftiofur; CEFO = cefotaxime; CEFR = ceftriaxone; CEFX = cefoxitin; CEPH = cephalothin; GENT = gentamicin; KANA = kanamycin; STREP = streptomycin; SULF = sulfisoxazole; SUMT= sulfamethoxazole; TMSX = trimethoprim–sulfamethoxazole; Ind = individual; *E. coli* = *Escherichia coli*; N/R = not reported.

References

[1] Waldner, C.L., Gow, S., Parker, S., Campbell, J.R. Antimicrobial resistance in fecal *Escherichia coli* and *Campylobacter* spp. from beef cows in western Canada and associations with herd attributes and antimicrobial use. *Can. J. Vet. Res.* 2019, 83, 80-89.

[2] Markland, S., Weppelmann,T.A., Ma, Z., Lee, S., Mir, R.A., Teng, L., Ginn, A., Lee, C., Ukhanova, M., Galindo, S., Carr, C., DiLorenzo, N., Ahn, S., Mah, J-H, Kim, H-Y, Mai, V., Mobley, R., Morris, J.G., Jeong, K.C.C. High prevalence of cefotaxime re-sistant bacteria in grazing beef cattle: a cross sectional study. *Front. Microbiol*. 2019, 10, 176 DOI:https://doi.org/10.3389/fmicb.2019.00176.

[3] Beef 2007–08 Antimicrobial Drug Use and Antimicrobial Resistance on U.S. Cow-calf Operations, 2007–08. Available online: https://www.aphis.usda.gov/animal_health/nahms/beefcowcalf/downloads/beef0708/Beef0708_ir_Antimicrobial_1.pdf (ac-cessed on 27 May 2023).

[4] Gow, S.P., Waldner, C.L. Antimicrobial resistance and virulence factors stx1, stx2, and eae in generic *Escherichia coli* isolates from calves in western Canadian cow-calf herds. *Microb. Drug Res.* 2009, 15, 61-67. https://doi-org.cyber.usask.ca/10.1089/mdr.2009.0860

[5] Carson, C.A., Reid-Smith, R., Irwin, R.J., Martin, W.S., McEwen, S.A. Antimicrobial resistance in generic fecal *Escherichia coli* from 29 beef farms in Ontario. *Can. J. Vet. Res.* 2008, 72, 119-128.

[6] Gow, S.P., Waldner, C.L., Rajic, A., McFall, M.E., Reid-Smith, R. Prevalence of antimicrobial resistance in fecal generic *Escherichia coli* isolated in western Canadian beef herds. Part II — Cows and cow-calf pairs. *Can. J. Vet. Res.* 2008, 72, 91-100.
